# Late Acheulian stone-working by the riverbank: Patterns of continuity and change reflected in Jaljulia lithic assemblages, Israel

**DOI:** 10.1371/journal.pone.0338540

**Published:** 2025-12-29

**Authors:** Tamar Rosenberg-Yefet, Aviad Agam, Ella Assaf, Bar Efrati, Yafit Kedar, Vlad Litov, Maayan Shemer, Ran Barkai

**Affiliations:** 1 Zinman Institute of Archaeology, University of Haifa, Mount Carmel, Haifa, Israel; 2 Department of Archaeology and Near Eastern Cultures, Tel Aviv University, Tel Aviv, Israel; 3 School of Archaeology, University of Oxford, Oxford, United Kingdom; 4 Department of Anthropology, Yale University, New Haven, Connecticut, United States of America; 5 Department of Anthropology, University of Connecticut, Charles Lewis Beach Hall, Storrs, Connecticut, United States of America; Universita degli Studi di Ferrara, ITALY

## Abstract

The Lower Paleolithic Late Acheulian marks an exceptional phase in human cultural evolution, encompassing notable transformations and innovations across Africa and Western Eurasia alongside the persistence of well-practiced Acheulian modes of adaptation. Lithic transformations mentioned here include innovative stone-working technologies such as prepared cores, Quina-like scrapers and possible origin of systematic blade production. These innovations provide a glance into potential changes in technological organization of lithic production that might reflect innovative modes of adaptation oriented towards changes in economy, environment and world-views of these early hominin groups. The open-air, Late Acheulian site of Jaljulia makes a significant contribution to the study of this transformative phase at the very end of the long Acheulian tradition in the Levant. The site was excavated to a relatively large extent (ca. 80m^2^) and the excavation yielded rich lithic assemblages of typical Late Acheulian technological components from several localities, dated to ca. 500–3/200 ka. The lithic assemblages are mostly dominated by flake-production, flake-tools, and numerous Handaxes. This paper presents the comprehensive analyses of the flint assemblages from five Jaljulia localities (Localities A–E). The results presented and discussed here are intriguing, as all five assemblages encompass components that could be regarded as forbearers of post-Acheulian industries. The use of prepared cores might signal an early appearance of the Middle Paleolithic Levallois concepts, and in order to stress this point, these cores are termed here “proto-Levallois”. Quina-like scrapers and blades are prominent, possibly reflecting the early adoption of technologies that became more common in the post-Acheulian, Acheuleo-Yabrudian cultural complex of the Levant. Based on this remarkable lithic repertoire, this paper discusses possible patterns of continuity and change in lithic production from the Late Acheulian to later industries and suggests refinements to the reduction trajectories variability.

## Introduction

The Acheulian Cultural Complex (hereafter Acheulian), which emerged approximately 1.9–1.8 million years ago (Mya), achieved an extensive geographic distribution across Africa and Eurasia during the Early and Middle Pleistocene [1–3]. Within this broader cultural and evolutional context, the Levantine Lower Paleolithic is primarily associated with the Acheulian cultural tradition, spanning from around 1.4 to 0.4/0.2 Mya [4–7].

Following their dispersals out of Africa, Acheulian populations exhibited exceptional ecological plasticity, successfully colonizing diverse biomes and climatic zones throughout the Old World [8–11]. This adaptive success was underpinned by behavioral flexibility, dietary breadth, and efficient exploitation of local faunal resources, enabling long-term survival under fluctuating environmental conditions [12,13,3]. In the Levant, a corridor linking Africa and Eurasia, Acheulian hominins are purely documented since no human remains have been recovered from Late Acheulian sites in the Levant. The only hominin fossils in the region are attributed to the Early Acheulian at Ubeidiya and to the Acheulo-Yabrudian Cultural Complex (AYCC) [14–16]. Subsistence strategies in the Levantine Acheulian reflect recurrent engagement with large terrestrial herbivores, whose acquisition and processing provided critical caloric and nutritional returns [17–20]. Zooarchaeological evidence, including cut-marked bones and patterned carcass exploitation, indicates that Acheulian hominins often had primary access to prey and likely engaged in organized hunting as well as opportunistic scavenging [21–24]. Large mammals such as proboscideans and ungulates formed the energetic backbone of these early human groups, with a single *Palaeoloxodon antiquus* providing sustenance for extended periods [25,26]. Beyond their nutritional role, such prey may also have held perceptual or symbolic significance, reflecting early ontological relationships between hominins, animal worlds, and the broader environment [27–29].

The late phase of the Acheulian (500–300 ka in the Levant; for other regions, see [15] is characterized by an accelerated pace of innovation incorporated into traditional lithic toolkits, while still exhibiting a strong continuity of cultural practices [15,30–34]. In addition to transformations in lithic technologies, the Acheulian, mostly the late phase of it, encompasses some significant processes and adaptations of human behavior, including: 1) the use of a wide spectrum of natural elements for dietary purposes (animals and plants), indicating high levels of familiarity with the local environment and the different ecotones as well as extensive traditional ecological knowledge [4,9,18,35–39], coupled by dependency on calories induced from protein and fat obtained from large and medium sized game [9,39–43]. 2) the beginning of controlled use of fire [44–46]. 3) the conduction of activities involving the cooperation of other group members (e.g., giant core technologies, hunting and processing mega-herbivores) [27,47–49]. 4) sophisticated stone acquisition and selectivity strategies to accommodate specific technological needs [50–53] 5) the use of soft hammer [48,54,55], 6) The appearance of early signs of cumulative culture [56–58].

In the Levant, this period coincides with paleoenvironmental changes, including shifts in mammalian communities that were primarily driven by extinctions rather than species replacement [25,43,58,59].

Acheulian core technology is mostly oriented towards the production of flakes in different dimensions and comprises of several core technologies, including cores with one, two or multi striking platforms; globular cores; prepared cores (also termed hierarchical cores); discoid cores and cores-on-flakes [e.g., 7,60–62]. Additionally, at some sites, specific core technologies appear alongside the rest of the categories described above, such as giant core technology, Acheulian micro-flakes, and handaxes with preferential flake scars [e.g., 56,60–65]. Of note are cores resemble the Middle Paleolithic Levallois ones, documented at several late Lower Paleolithic assemblages in a wide geographic dispersal, termed here Proto-Levallois cores [7,66–74], but see White and colleagues [75] suggesting these are not Levallois related. Of special note are very small flakes detached from cores-on-flakes and from regular cores. These were intentionally produced as precise, single-use tools tailored for specific butchery tasks, reflecting a deliberate and skilled approach to resource utilization [76–80].

Thus, Late Acheulian flake production was aimed at providing sets of blanks in different sizes and morphologies to be subsequently shaped into tools or, in other cases, to be utilized with no further shaping [e.g., 80,81]. These commonly dominate Acheulian lithic assemblages, in addition to less frequent but much more conspicuous tool-types like handaxes, also referred to as bifaces or Large Cutting Tools (LCTs) [e.g., 6,7,61,77,78,82–88], Cleavers, Chopping-tools [89] and stone balls (spheroid/polyhedron) [90,91].

The Acheulian handaxe appears in a vast geographical and chronological range, spanning ca. 1–1.5 million years and found in lithic assemblages in Africa, Europe, and Asia [e.g., 12,60,61,63–98]. While in Europe, biface manufacture continued into the Middle Paleolithic, in the Levant handaxes disappeared altogether from post-Acheulian, Middle Paleolithic industries [5,99].

The Lower Palaeolithic in the Levant ends with a local, regionally limited culture known as the Acheulo-Yabrudian Complex (AYCC). Chronologically, the AYCC appears to follow the Late Acheulian in most cases, although some temporal overlap may have occurred, and the nature of the relationships between these different modes of human adaptation remains unclear.

The AYCC is well stratified, occupying a post-Acheulian and pre-Mousterian position at sites such as Qesem Cave, Tabun Cave, Yabrud I, Hayonim Cave and Zuttiyeh [8,100–104]. Dating analysis across a variety of Levantine sites such as ESR, U-series, U-Th, and TL places the AYCC between ~400–200 kyr [104–107].

The AYCC is unique in its lithic characteristics, comprising three co-occurring industries, with the primary distinction between them based on the relative abundance of specific tool types within each assemblage. Acheulo-Yabrudian industry – a flake oriented tradition dominated primarily by handaxes; Yabrudian industry – characterized by Quina and demi Quina scrapers; Amudian industry – focused on the production of laminar blanks and tools [100,107–112].The current paper presents the techno-typological characteristics of the lithic assemblage of five localities excavated at Jaljulia, whereas the assemblage of another locality (G) excavated during the second campaign at the site, is currently under study and will be published in the future. The analyses include technological aspects, detailing the different knapping trajectories used on site, including lithic recycling and the use of advanced core technologies such as the proto-Levallois method. Differences between the assemblages are discussed as inter-site variability, considering also the chrono-stratigraphy of the site. These are used to assess aspects of cultural continuity both within the Late Acheulian and into post-Acheulian industries, alongside variability in lithic production and the levels of investment in new and innovative lithic trajectories.

## Materials and methods

### The late Acheulian site of Jaljulia

**Fig 1:**
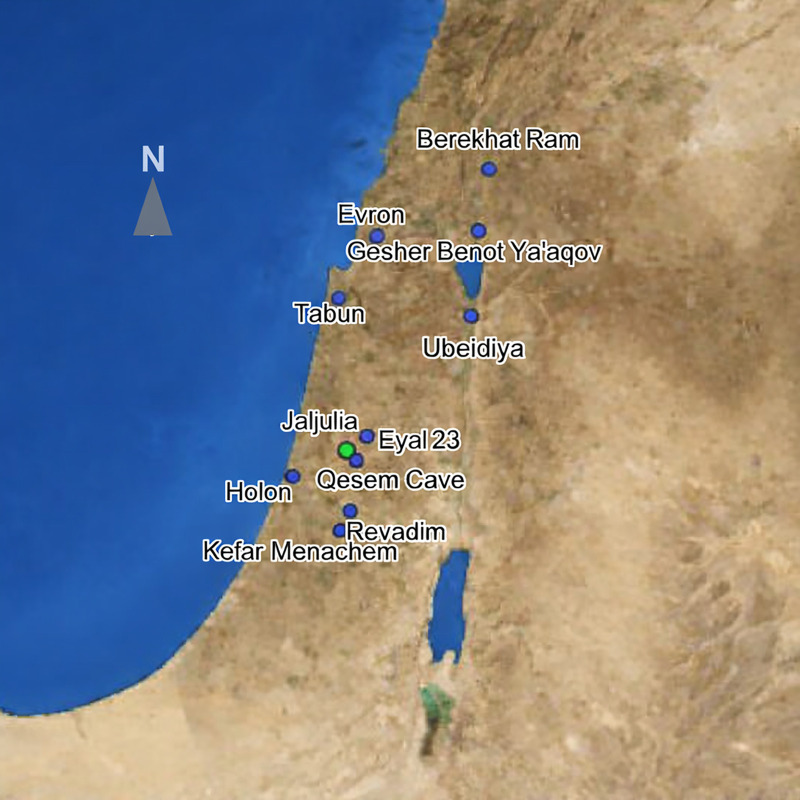
A map showing the location of Jaljulia and other relevant sites mentioned in the text. All but Qesem Cave represent Acheulian sites, Tabun represent both Acheulian and Achelo-Yabrudian layers.

**Fig 2:**
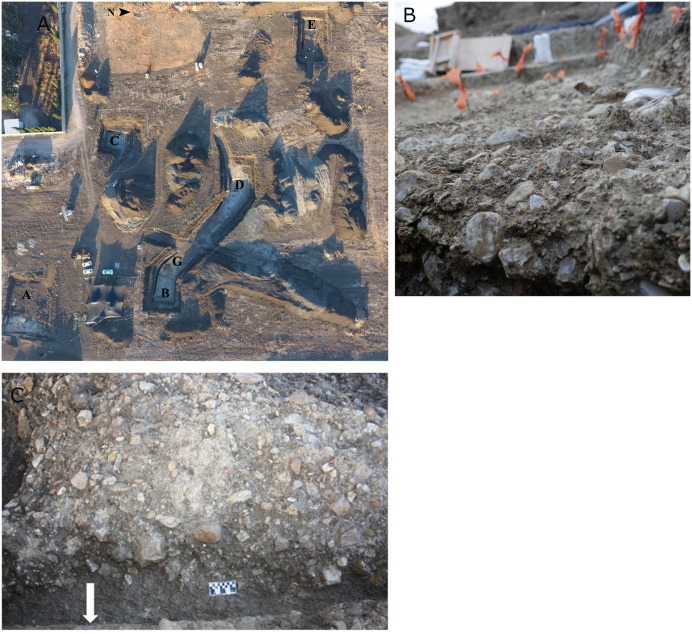
A: Excavated localities A–E and G; B: close-up of locality B; C: close-up of locality A.

**Fig 3:**
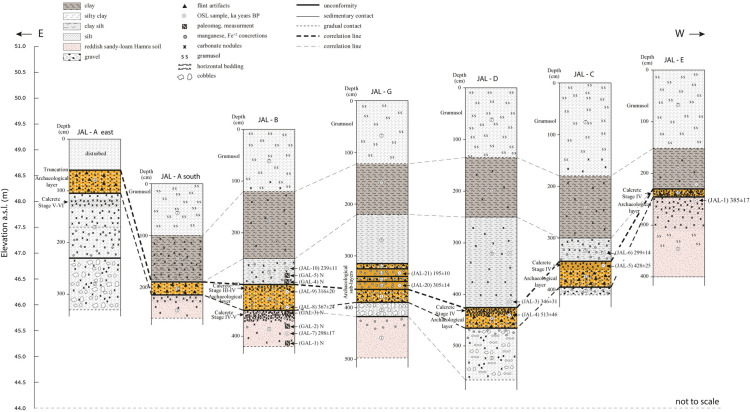
Stratigraphy of the site of Jaljulia as displayed in all sampled sections, as well as the sampling locations for absolute dating and for paleomagnetic stratigraphy. Note, that locality G has not yet been analyzed and therefore is not part of the current lithic study [113].

The site is located in the central coastal plain of Israel, on an ancient tributary of Wadi Qana (Fig 1). It is situated approximately six km south of the late Acheulian site of Eyal and six km northwest of the Acheulo-Yabrudian site of Qesem Cave (Fig 1) [113]. Approximately 80 m² of what is estimated to be more than 1 hectare of Late Acheulian deposits were excavated during 2016–17, in the framework of a salvage excavation by the Israel Antiquities Authority in collaboration with the Department of Archaeology at Tel Aviv University, prior to a large housing project [113]. The excavation was conducted in six localities (Localities A–E, G; Fig 2A), with Late Acheulian deposits covered by 1.5–5.5 m of sediments. With the exception of Area E, the archaeological artifacts in all localities were deposited on top of a dense and partly concreted conglomerate layer, rich in natural flint nodules (Unit 4, Fig 3). Comprehensive geomorphological and site formation studies have demonstrated that Jaljulia was an active floodplain at the time of human activity, possibly associated with W. Qanah, nowadays a seasonal stream, flowing ca. 1 km south of the site [113]. The presence of manganese and iron concretions alongside carbonate coating of some of the nodules indicates a dynamic marshland or wetland environment that was active over a long period of time; thus, supporting recurrent visits by ancient human groups throughout the Late Acheulian. This marshland environment mostly dried up by ca. 300 ky BP as reflected by the presence of a developed calcrete soil on top of Unit 4, leaving a much smaller fluvial presence limited to the immediate surroundings of Area G (Fig 2; [113]). Locality E is unique, as it was deposited on a low Hamra hill, overlooking the fluvial plain in which the other localities were situated.

The excavation revealed archaeological layers rich in flint artifacts, whereas faunal preservation was poor, as few (n = 38), highly fragmented animal bones were found only in one of the excavated localities (Locality D, 116). The low representation of animal bones is probably due to site formation processes that eliminated most of the organic archaeological material. 6–25 m² were excavated in each of the localities, following a grid of 1 m² squares. Each 1 m^2^ square was further divided into four sub-units, and the excavation was conducted in 5–10 cm deep controlled spits. All excavated sediments were dry-sieved using a five mm mesh.

Samples for absolute ESR and Luminescence dating were collected from localities B, C, D, E, and G, indicating a general chronological frame of 500–300/200 ka BP for the human activity at the site, with each locality potentially reflecting a different activity phase. The oldest Acheulian occupations were discovered in localities D (~513−417 ka) and C (~444−428 ka), followed by B (~367−316 ka). Locality G, in which five distinctive archaeological units were identified, yielded an overall younger set of dates, though these assemblages will be published in a separate paper (~310−195 ka). No ages were obtained for localities A and E (116; Fig 3). The preliminary ascription of Jaljulia to the Late Acheulian was based on characteristics of the lithic assemblages, such as the presence of relatively small handaxes and vast and diversified flake production techniques. Geological studies focused on site formation and post depositional processes, alongside faunal analysis and preliminary lithic studies, indicate that the site of Jaljulia was located in the vicinity of an old stream-bed, providing a stable source of fresh water, which likely supported, at least seasonally, lush vegetation and animal groups [113]. The wadi bed contained numerous flint nodules that were carried by the stream from primary sources in the Samaria hills to the east. It is therefore estimated that the site of Jaljulia was frequented by Late Achaeulian group/s over the long period of time indicated by absolute dating results and spanning several hundred millennia and thus reflects a long persistency of human presence at this Paleolithic landscape.

Jaljulia is located in a Turonian (Upper Cretaceous) terrain, an area marked by numerous exposures of flint-rich limestone associated with the Bi’na Formation. The archaeological layer at the site was deposited just above an ancient conglomerate, interpreted as a remnant of a floodplain once linked to Wadi Qanah, a stream that flowed in from the east. In locality A, an old streambed was exposed, likely representing a former channel of Wadi Qanah. The current course of the wadi lies about 100 meters south of the site. Both the ancient and modern wadi paths are rich in flint nodules of different shape and size, which originated from a variety of geological sources. These nodules, found in the site conglomerate and along Wadi Qanah, were probably a key source of raw material used by the Jaljulia tool makers [52].

A recent study evaluated patterns of flint procurement and exploitation at Jaljulia, indicating that locally available flint types, i.e., flint nodules that could be found in the riverbed underlying the archaeological layers, were indeed commonly used at the site, further stressing its unique setting and potential attractiveness to hominin groups at a source for raw material procurement. Selectivity in flint types was observed for particular lithic production trajectories, such as bifaces and prepared cores [52]. For example, in a recent study of flint types across four artifact groups, a general sample, bifaces, “regular” cores, and prepared cores, clear patterns of raw material selection emerged. While local Turonian flint dominates the general assemblage, selective use of specific flint types was evident. Brecciated flint was common in handaxes and discoid cores, whereas fine-textured, homogeneous flint appeared more often in proto-Levallois and prepared cores, suggesting a preference for higher-quality materials when greater control over reduction was needed. Intriguingly, this study showed similar selection patterns in the oldest locality, Locality D (ca. 500 ka BP) and one of the youngest localities, Locality B (ca. 300 ka BP), suggesting that Jaljulia’s inhabitants were consistent regarding the flint types used for particular trajectories [52]. In addition, examining the flint types of scrapers revealed that 58% (n = 125) of the scrapers were made of Campanian flint of the Mishash Formation. While Campanian flint types are locally available, several distinctive types have not yet been discovered in either primary or secondary depositions in the vicinity of the site. A potential source for the nonlocal flint types may have been located approximately 20–25 km east of Jaljulia in the Samaria hills, making this flint selectivity even more interesting [114].

### The archaeological context

The lithic assemblages presented in this study originated from five localities of excavation at the site, labeled Localities A–E. For a detailed description of all localities of excavation, see [113].

Locality A (Fig 2C) is the southern-most locality excavated in Jaljulia. Locality B (Fig 2B), is at the eastern part of the site, and is the densest in terms of lithic findings. Locality C is located in the western part of the site. It yielded relatively low numbers of finds and was suggested to represent the possible margins of the riverbed. Locality D is the largest of the five localities presented here and is located at the center of the site. This was also the only excavated locality to reveal fauna remains, even if very scarce. The density of lithic findings in locality D is average compared to the other localities [113]. Locality E is the northeastern most locality of excavation, and is unique by containing a thin archaeological horizon (Table 1) deposited directly on Hamra soil, rather than on ancient fluvial deposits. This locality was suggested to represent isolated activities conducted on a low hill overlooking the wadi. The best state of preservation was observed at the northeastern part of the site, in localities B, D, and E. Artifacts in localities B and D were mostly found in horizontal orientation.

**Table 1:** Excavation and assemblage’s details [113].

Locality	Excavated area (m^2^)	Average thickness of the archaeological horizon (m)	Estimated volume of excavated sediments (m^3^)	Size of lithic assemblage (N)	Average density for cubic meter (N)
Area A	11	0.30	3.3	12,011	3640
Area B	6	0.35	2.1	16,413	7816
Area C	13	0.20	2.6	6,840	2631
Area D	16	0.30	4.8	24,126	5028
Area E	12	0.08	0.96	5,247	5971

### Methods

The qualitative and quantitative analysis presented here follows the principles of the *chaîne opératoire* approach. As such, it is based on the identification of the different phases of lithic reduction and maintenance [e.g., 115–117]. All knapped lithic finds, including those smaller than 1.5 cm, were analyzed in the current study, following the approach and basic definitions used previously for the analysis of Lower Paleolithic assemblages from the Levant [e.g., 6,7,27,29,53,75,77,118,119]. A detailed description of the lithic categories appears in S1 File.

## Results

### Techno-typological characteristics of the lithic assemblages

General breakdowns of the assemblages analyzed in this study, counting a total of 65,174 items, are presented in Tables 2 and 3. Although presenting some variability in the abundance of certain categories, all assemblages contain similar techno-typological components. Localities A and E present the highest frequencies of debris (i.e., chips, chunks; ca. 70%), whereas Localities B and D present the lowest (ca. 57%; Tables 2 and 3). Shaped items constitute 9.2% of each assemblage on average, but the differences between localities can be significant. Thus, in Localities D and B, shaped items constitute 16.1% and 12.4% of the assemblage, respectively, whereas Localities A and C present milder abundance (7.5% and 6.6%, respectively) and in Locality E, shaped items represent only 3.4% of the assemblage.

**Table 2:** General breakdown of the lithic assemblage of localities A–C.

Locality	Locality A no.	Locality A % of *débitage* and shaped items	Locality A % of total assemblage	Locality B no.	Locality B % of *débitage* and shaped items	Locality B % of total assemblage	Locality C no.	Locality C % of débitage and shaped items	Locality C % of total assemblage
Primary element flake (PE flake)	536	14.6%	4.5%	1055	15%	6.41%	568	23%	8.3%
Primary element blade (PE blade)	13	0.4%	0.1%	44	1%	0.27%	15	1%	0.2%
Non-modified base flake	804	21.9%	6.7%	1320	19%	8.02%	605	25%	8.8%
Modified base flake	384	10.5%	3.2%	724	10%	4.40%	187	8%	2.7%
Blade	21	0.6%	0.2%	38	1%	0.23%	10	0%	0.1%
Lip flake	57	1.6%	0.5%	127	2%	0.77%	10	0%	0.1%
Prepared core flake	21	0.6%	0.2%	99	1%	0.60%	5	0%	0.1%
Core trimming element (CTE)	193	5.3%	1.6%	355	5%	2.16%	121	5%	1.8%
Core	431	11.8%	3.6%	629	9%	3.82%	281	11%	4.1%
Core on flake	178	4.9%	1.5%	195	3%	1.19%	122	5%	1.8%
Recycled products	105	2.9%	0.9%	206	3%	1.25%	69	3%	1.0%
Shaped items	900	24.5%	7.5%	2032	29%	12.35%	450	18%	6.6%
Tool waste	25	0.7%	0.2%	229	3%	1.39%	21	1%	0.3%
**Sum of *débitage* and shaped items**	**3668**	**100.0%**	**30.5%**	**7053**	**100%**	**42.86%**	**2464**	**100%**	**36.0%**
Chunk flake	1762		14.7%	**3735**		22.70%	1739		25.4%
Chunk	1944		16.2%	3094		18.80%	1211		17.7%
Chip	3974		33.1%	1820		11.06%	1131		16.5%
Micro flake	659		5.5%	745		4.53%	287		4.2%
Raw material	4		0.0%	7		0.04%	8		0.1%
**Total**	**12011**		**100.0%**	**16454**		**100.00%**	**6840**		**100.0%**

**Table 3:** General breakdown of the lithic assemblage of localities D and E.

Locality	Locality D No.	Locality D % of *débitage* and shaped items	Locality D % of total assemblage	Locality E No.	Locality E % of *débitage* and shaped items	Locality E % of total assemblage
Primary element flake (PE flake)	1448	14%	6.00%	379	21.7%	6.61%
Primary element blade (PE blade)	29	0%	0.12%	6	0.3%	0.10%
Non-modified base flake	1499	14%	6.21%	523	29.9%	9.12%
Modified base flake	783	8%	3.24%	96	5.5%	1.67%
Blade	25	0%	0.10%	4	0.2%	0.07%
Lip flake	90	1%	0.37%	1	0.1%	0.02%
Prepared core flake	61	1%	0.25%	4	0.2%	0.07%
Core trimming element (CTE)	521	5%	2.16%	99	5.7%	1.73%
Core	1265	12%	5.24%	279	16.0%	4.87%
Core on flake	362	3%	1.50%	64	3.7%	1.12%
Recycled products	249	2%	1.03%	80	4.6%	1.40%
Shaped items	3891	38%	16.12%	194	11.1%	3.38%
Tool waste	137	1%	0.57%	20	1.1%	0.35%
**Sum of débitage and shaped items**	**10360**	**100%**	**42.92%**	**1749**	**100.0%**	**30.51%**
Chunk flake	5259		21.79%	1269		22.14%
Chunk	2616		10.84%	812		14.16%
Chip	4814		19.95%	1535		26.77%
Micro flake	1065		4.41%	282		4.92%
Raw material	22		0.09%	86		1.50%
**Total**	**24136**		**100.00%**	**5733**		**100.00%**

Another example is the abundance of non-modified base flakes, which is relatively high in Localities A (21.9%), C (25%), and E (29.9%) compared to Localities B (19%) and D (14%).

These differences may reflect variability among the five localities, potentially attributable to differing depositional conditions (e.g., Locality E), variations in the intensity of activities conducted at each locality (Table 1), or distinct emphases in lithic reduction strategies. Nevertheless, the similarities seem to be greater than the differences, as the flint assemblages from all localities align to late Acheulian lithic technological characteristics. Following is a description of the primary techno-typological traits of the Jaljulia assemblages and their degree of abundance in each locality.

#### Cores.

The vast majority of the cores were aimed at producing items of flake proportion (Table 4). Nevertheless, laminar cores and combined cores of both flakes and blades are present, albeit in very small numbers.

**Table 4:** Breakdown of core types.

Type of core	Locality A no.	Locality A %	Locality B no.	Locality B %	Locality C no.	Locality C %	Locality D no.	Locality D %	Locality E no.	Locality E %
Flake cores	356	82.6	465	73.9	149	53	925	73.1	161	57.7
Single striking platform	159	36.9	241	38.3	54	19.2	374	29.6	49	17.6
Two striking platforms	135	31.3	141	22.4	64	22.8	326	25.8	71	25.4
Multiple striking platforms	62	14.4	83	13.2	31	11	225	17.8	41	14.7
FL/BL cores	3	0.7	1	0.2	1	0.4	9	0.7	0	0
Laminar cores	0	0	2	0.3	4	1.4	5	0.4	0	0
Prepared cores	21	4.9	105	16.7	15	5.3	138	10.9	13	4.7
Prepared cores (second type)	13	3	19	3	9	3.2	54	4.3	9	3.2
Proto-Levallois cores	5	1.2	49	7.8	4	1.4	35	2.8	1	0.4
Discoidal cores	3	0.7	37	5.9	2	0.7	49	3.9	3	1.1
Core fragment	17	3.9	28	4.5	48	17.1	128	10.1	32	11.5
Tested core	34	7.9	24	3.8	64	22.8	59	4.7	73	26.2
Varia	0	0	4	0.6	0	0	1	0.1	0	0
Total	431	100	629	100	281	100	1265	100	279	100

**Fig 4:**
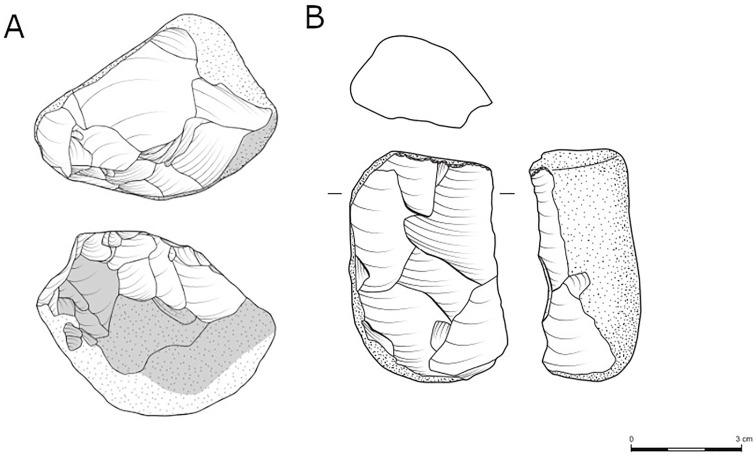
Single platform cores from locality B.

**Fig 5:**
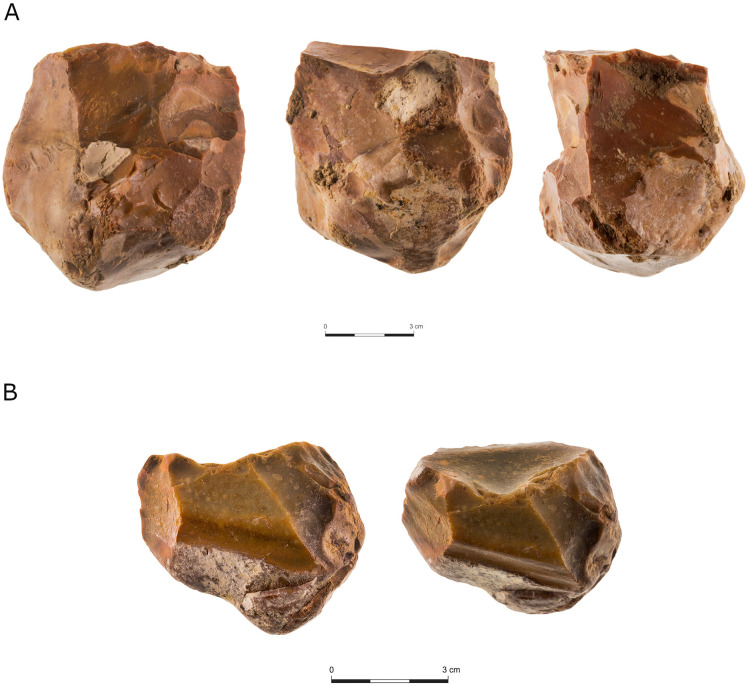
Single-platform cores from locality B.

“Regular” flake cores represent the most dominant reduction sequence in all assemblages. As will be demonstrated later, both the CTE’s and the number of flakes with modified bases (between 8%–10% of the *débitage* and shaped items for all assemblages), indicate that these cores were subjected to some degree of core preparation, maintenance, and rejuvenation. Nevertheless, the degree of dominance varies between localities, as in localities C and E, these cores represent half of all cores, whereas in localities A, B and D they constitute much higher percentages (Table 4). Within this category, cores with a single striking platform (Figs 4 and 5) are the most common, followed by two-striking platforms and multiple-striking platforms cores.

Tested pebble/cobbles, probably aimed at testing the quality of the flint and therefore representing some initial stage of material selectivity also show variability in abundance between assemblages, with notably high frequencies in Localities C, E (ca. 23% and 26% of the cores, respectively; Table 4).

**Fig 6:**
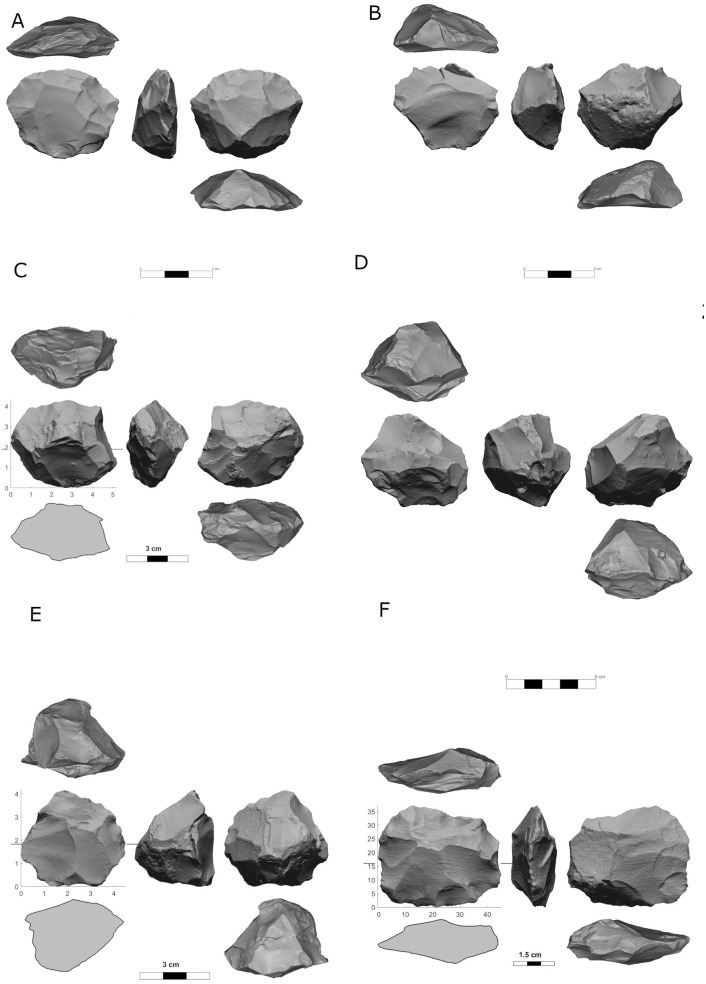
Prepared cores from locality B: proto-Levallois (A, B), discoid cores (C, D) and prepared cores (general) (E, F).

Prepared cores also present an intriguing variability in abundance, showing the highest frequencies in Localities B (Fig 6) and D (16.7% and 10.9% of the cores, respectively), while appearing in relatively modest numbers in Localities A, C and E (ca. 5% of the cores, Table 4).

Furthermore, there is a noted variability in the abundance of prepared core subtypes between localities. For locality B, a further analysis was conducted on all but 13 prepared core fragments, as addressed later in the discussion section. These were selected since locality B was the first to be sorted out of all five localities. All Proto-Levallois cores are intended to be analyze in the near future. For a detailed analysis of prepared cores from the site locality see Rosenberg-Yefet et al. 2022 [29].

**Fig 7:**
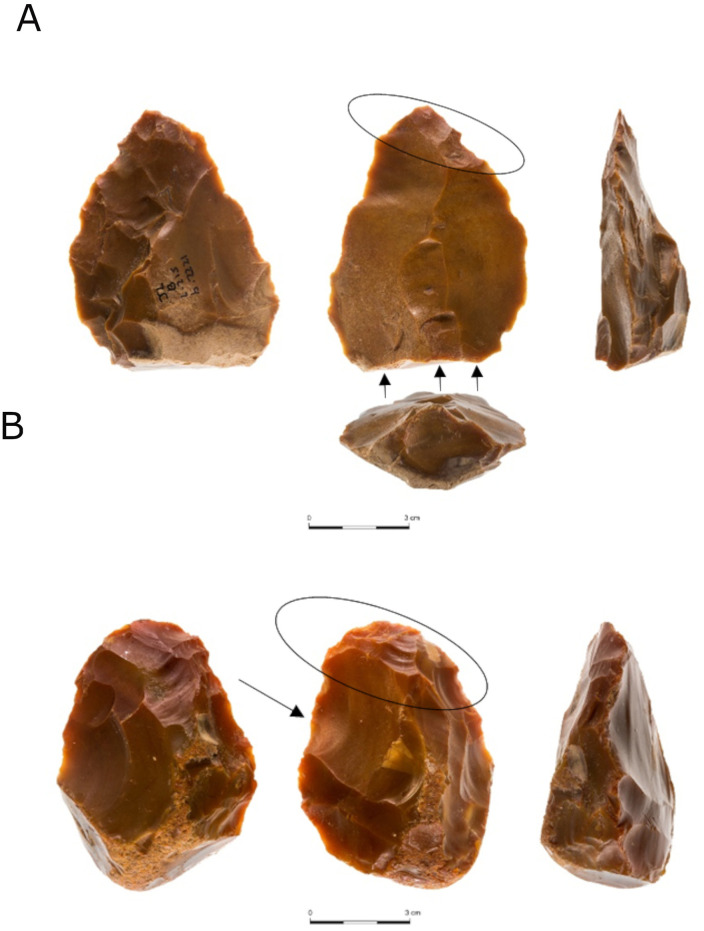
A: A handaxe transformed into a prepared core from Jaljulia. Evidence for the first life-cycle of the item as a handaxe is found on the striking platform of the core (left image), and only minimally on the production platform (middle image), characterized by a series of flat multi-directional removals. Two main large items (one with blade proportion) and one small flake were removed from the handaxe (marked by arrows). The scars can be seen to have cut the previous bifacial shaping scars at the tip of the handaxe (marked with a circle). B: A handaxe with isolated flake removal from Jaljulia. Both faces are characterized by flat multi-directional removals. Additionally, a bifacial ridge is clearly observed on both lateral edges. One large flake was removed from the handaxe (marked by an arrow). The scar can be seen to have cut the previous bifacial shaping scars (marked with a circle).

An additional group of items, aiming at the production of flakes using a knapping method bearing some similarity to the Proto-Levallois cores, is the handaxes with preferential flake removals (n = 14, Table 10; Fig 7). In these items, the knappers took advantage of the convexities that characterize bifaces, enabling the detachment of predetermined blanks with minimal preparatory steps. The detachment of predetermined blanks from existing handaxes has been documented in many late Lower Paleolithic sites around the world—in Africa [68], the Levant [7,56,65,78,120], Europe [121,122], Caucasus [73], and Asia [123–125].

**Table 10:** Composition of biface types from all localities.

	Locality A no.	Locality A %	Locality B no.	Locality B %	Locality C no.	Locality C %	Locality D no.	Locality D %	Locality E no.	Locality E %
**Type**	No.	%	No.	%	No.	%	No.	%	No.	%
**Biface**	6	15%	11	9%	0	0%	16	9%	3	12%
**Biface fragment**	1	2%	2	2%	0	0%	9	5%	0	0%
**Handaxe**	18	44%	63	50%	2	22%	101	55%	10	38%
**Handaxe fragment**	0	0%	14	11%	2	22%	13	7%	0	0%
**Small handaxe**	3	7%	7	6%	2	22%	26	14%	0	0%
**Core-Handaxe**	3	7%	5	4%	0	0%	4	2%	5	19%
**Roughout**	10	24%	11	9%	3	33%	15	8%	8	31%
**Hanaxes with preferential flake removals**	0	0%	14	11%	0	0%	0	0%	0	0%
**Total**	41	100%	127	100%	9	100%	184	100%	26	100%

Handaxes with preferential removal are generally larger than the general sample of handaxes from locality B. The preferential flake/s were most frequently detached from the proximal end of the handaxe (the base) towards the tip and more rarely from other parts. For a detailed description of these items from locality B, see 13, and similar items from the other localities still awaits analysis.

**Fig 8:**
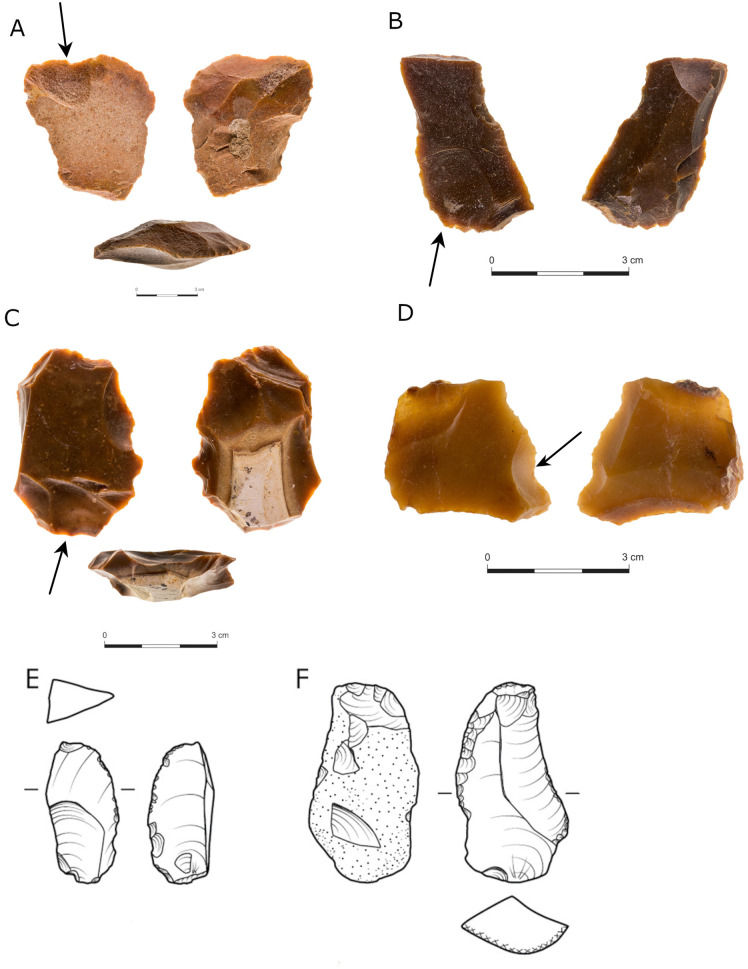
Cores on flakes from locality B, ventral single (A, B, D) and ventral multiple (C). Cores on flake from locality D, ventral single (E, F). Note the arrow is pointing to the place where the items were removed from the parent flake.

Cores-on-flakes comprise between 3% to 4.9% of the *débitage* in each assemblage (Tables 2 and 3). In all localities, most removals focus on the ventral face, demonstrating either a single (Fig 8A, 8B and 8D), or multiple removals (Fig 8C) (Table 5). COF presenting dorsal removals are also present in all localities, however they are less common in locality B (9.7% of the category; Table 5). A combination of both ventral and dorsal removals was also found in notable numbers (Table 5). Only in Locality B COFs with multiple removal scars are more common than COFs with a single removal scar (Table 5).

**Table 5:** Breakdown of COFs types from all localities.

	Locality A no.	Locality A %	Locality B no.	Locality B %	Locality C no.	Locality C %	Locality D no.	Locality D %	Locality E no.	Locality E %
**Ventral Single**	57	32.0%	49	25.1%	51	41.8%	124	34.3%	26	40.6%
**Ventral Multiple**	40	22.5%	77	39.5%	24	19.7%	89	24.6%	18	28.1%
**Dorsal Single**	27	15.2%	7	3.6%	11	9.0%	54	14.9%	6	9.4%
**Dorsal Multiple**	3	1.7%	12	6.2%	6	4.9%	22	6.1%	3	4.7%
**Combined**	23	12.9%	39	20.0%	20	16.4%	62	17.1%	7	10.9%
**Varia**	28	15.7%	11	5.6%	10	8.2%	11	3.0%	4	6.3%
**Total**	**178**	**100.0%**	**195**	**100.0%**	**122**	**100.0%**	**362**	**100.0%**	**64**	**100.0%**

#### Core trimming elements (CTE).

CTEs comprise ca. 5% of the *débitage* in all assemblages (Tables 2 and 3). Excluding the *varia* category, simple ridges are the most common maintenance item in Localities B, C, and D, overshot items are common in all localities except Locality A, while core tablets are more frequent in localities A and E. While typically the term core tablet refers to a waste removed from blade cores, here we address it more generally as an item removed from a flake/blade core aiming at the renovation of the core striking platform. These items bears scars from removals that were made prior to its detachment, visible on the dorsal surface (on the items thickness).

**Fig 9:**
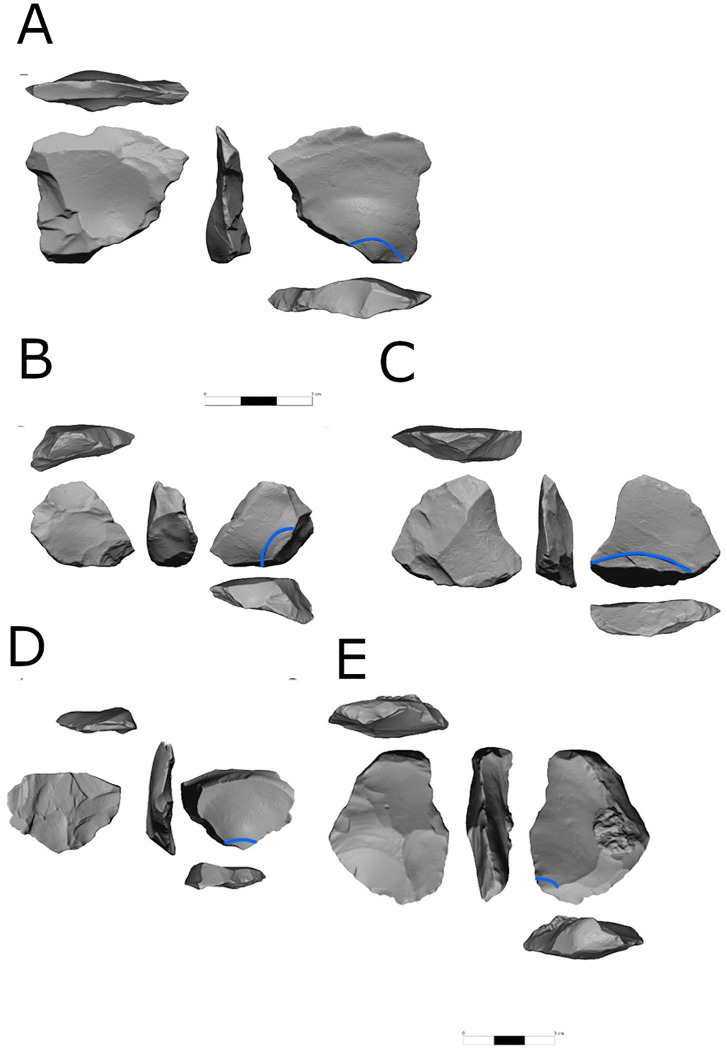
*Éclats débordant* CTEs from Jaljulia. The bulb of percussion is highlighted by a blue line. A–D: *Éclats débordant*; E: *Outrepassé*. Note that for this item, both the lateral and distal ridge of the core have been removed.

CTEs detouched from prepared cores are most common in Localities B and D (Fig 9), correlating to the high frequencies of prepared cores noted in these localities (Table 4), while quite rare in the other assemblages (Table 6). *Entame* flakes (flakes covered by 100% cortex; [31]) are minimally represented in all assemblages (Table 6), Core tablets are common in localities A and E (Table 6). Prepared core CTEs of locality B were further divided in a separate paper into sub-types [29]. Out of the 60 items defined as related to the prepared cores, 33.3% (n = 20) were defined as *éclats débordants* and the rest were defined in the more general definition of prepared core CTEs (see supplementary material for explanation of the difference between the definitions).

**Table 6:** Breakdown of CTE items from all localities.

	Locality A no.	Locality A %	Locality B no.	Locality B %	Locality C no.	Locality C %	Locality D no.	Locality D %	Locality E no.	Locality E %
**Core trimming elements**	**Total**	**%**	**Total**	**%**	**Total**	**%**	**Total**	**%**	**Total**	**%**
**Core Tablet**	52	26.9%	0	0.0%	7	5.8%	24	4.61%	19	19.2%
**Overshot**	7	3.6%	39	11.0%	15	12.4%	52	9.98%	7	7.1%
**Ridge**	16	8.3%	147	41.4%	23	19.0%	92	17.66%	13	13.1%
**Prepared Core**	3	1.6%	60	16.9%	7	5.8%	63	12.09%	0	0.0%
**Cortical flake (*Entame*)**	1	0.5%	7	2.0%	0	0.0%	9	1.73%	9	9.1%
**Varia**	114	59.1%	102	28.7%	69	57.0%	281	53.93%	51	51.5%
**Total**	**193**	**100.0%**	**355**	**100.0%**	**121**	**100.0%**	**521**	**100.00%**	**99**	**100.0%**

#### Technologically determined blanks.

**Fig 10:**
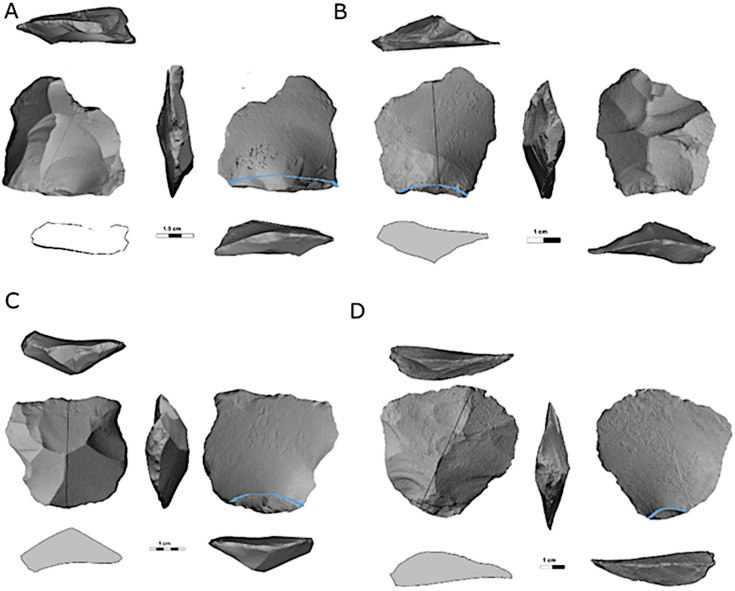
Flakes produced from prepared cores. The bulb of percussion is highlighted by a blue line.

***Flakes produced from prepared cores* (**Fig 10**):** These are flakes that were modified prior to their detachment from the core and therefore, carry characteristic scars on their dorsal surfaces. These items appear in low percentages in all assemblages—1% of the *débitage* in localities B and D, and even less in other localities (Tables 2 and 3). In Locality B, the vast majority of items demonstrate a faceted striking platform, followed by dihedral platforms. Punctiform, cortical, and *en chapeau de gendarme* striking platforms are less common (Table 7; Rosenberg-Yefet at al. 2022 [29]).

**Table 7:** Striking platform preparation of prepared core blanks from Locality B.

Type of striking platform	no.	%
Plain	11	11.1
Punctiform	2	2
Faceted	60	60
Dihedral	17	17
*En chapeau de gendarme*	3	3
Cortical	2	2
Undetermined	4	4
Total	99	100

**Fig 11:**
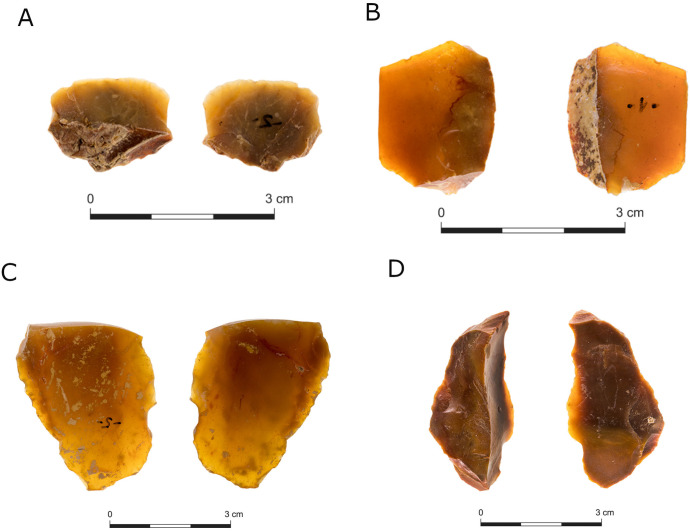
Blanks produced from COF, regular items (A–C) and lateral item (D). Note the patina differences between the two ventral faces of the item (D).

***Blanks produced from COF* (**Fig 11**):** Some of the COF represent cases of lithic recycling at Jaljulia and relates to a wider phenomenon of recycling at late Acheulian sites, which includes: systematic production of small sharp flakes from parent flakes (core-on-flake) [77,78,126–129]; the use of handaxes as cores for the production of predetermined blanks [7,56,120–122]; and the use of old patinated cores and flaked items for the production of new items [77,47,130–133].

Hundreds of products of COF were observed in all five assemblages, constituting 2% –4.9% of the *débitage* (Tables 2 and 3). All recycled products were further divided into categories based on the location from which they were detached on the parent flake, as termed by Agam and Barkai [77]. Excluding the varia category, the most common category in all localities is regular items (Table 8) (Fig 11A–11C), followed by lateral (Fig 11D) and reversed lateral items. Double ventral overshot are common only in locality C and less common in the other assemblages (Table 8). Double-bulb (DB) items are represented in small numbers in all assemblages (12.5%–16.5% of all recycled items; Table 8). Some of the items bear double patina, indication of the time that passed between the two production cycles, that of the parent flake and that of the recycled flake (Fig 11D). Use-wear and residue analyses of similar items from the sites of Revadim and Qesem cave indicated purposeful production of these sharp items, mostly oriented towards dedicated tasks within the butchery process [130,47]. It is reasonable to assume similar use of the Jaljulia recycled flakes, however the assessment of such a hypothesis awaits future analyses.

**Table 8:** Breakdown of blanks produced from COF from all localities.

	Locality A no.	Locality A %	Locality B no.	Locality B %	Locality C no.	Locality C %	Locality D no.	Locality D %	Locality E no.	Locality E %
**Products of Recyclyng**	**no.**	**%**	**no.**	**%**	**no.**	**%**	**no.**	**%**	**no.**	**%**
**Regular**	35	33.3%	85	41.3%	14	20.3%	72	28.9%	23	28.8%
**Lateral**	12	11.4%	34	16.5%	12	17.4%	30	12.0%	14	17.5%
**Reversed Lateral**	15	14.3%	12	5.8%	3	4.3%	21	8.4%	9	11.3%
**Double Ventral Overshot**	3	2.9%	5	2.4%	12	17.4%	0	0.0%	2	2.5%
**Varia**	24	22.9%	36	17.5%	18	26.1%	85	34.1%	22	27.5%
**DB Kombewa**	9	8.6%	18	8.7%	3	4.3%	17	6.8%	3	3.8%
**DB Lateral**	2	1.9%	5	2.4%	4	5.8%	6	2.4%	0	0.0%
**DB Reversed Lateral**	2	1.9%	0	0.0%	0	0.0%	1	0.4%	3	3.8%
**DB Tabun Snap**	1	1.0%	2	1.0%	1	1.4%	3	1.2%	0	0.0%
**DB Varia**	2	1.9%	9	4.4%	2	2.9%	14	5.6%	4	5.0%
**Total**	**105**	**100.0%**	**206**	**100.0%**	**69**	**100.0%**	**249**	**100.0%**	**80**	**100.0%**

**Fig 12:**
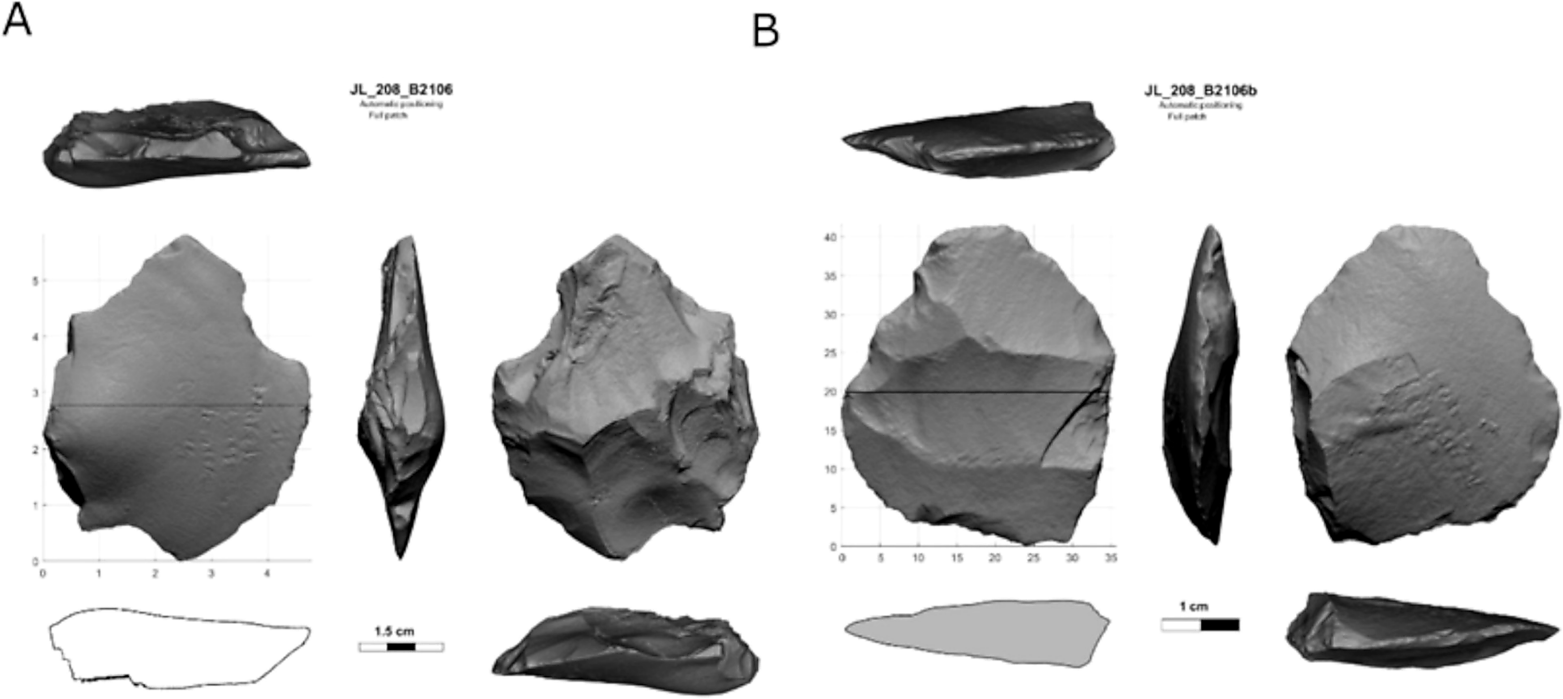
Flakes from locality B.

***Technologically undetermined blanks* (**Fig 12**):** Primary (cortical) flakes are most frequent in localities C (23%) and E (21.7%), while presenting lower frequencies (14%–15%) in all other localities (Tables 2 and 3). Primary blades are significantly less common, constituting1% or less of the *débitage* in each assemblage (Tables 2 and 3). MBFs (modified base flakes), reflecting modification done prior to detachment, constitute 8%–10.5% of the blanks in all localities excluding locality E (Tables 2 and 3). Flakes with a non-modified base are significantly more dominant in all assemblages constituting 19%–29% of the debitage in all localities except for Locality D, where a relatively lower percentage was noted (Tables 2 and 3). The appearance of flakes with some degree of modification of the base can be a further indication for core preparation of these regular cores, hinting for a more gradual process of the concept of prepared cores.

#### Shaped items.

**Fig 13:**
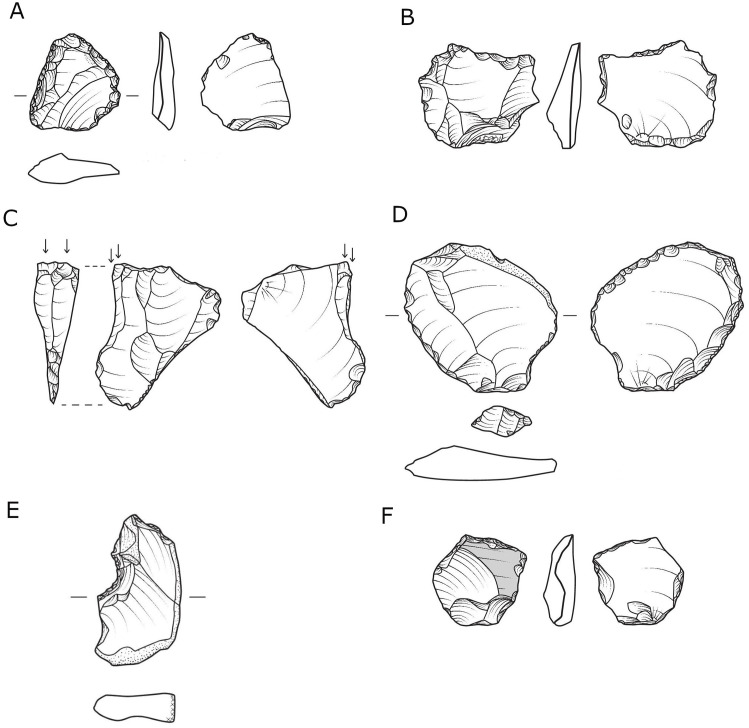
Shaped items from locality B: scraper (A, D), retouched flake (B), burin (C), notch (E), and truncation (F).

**Fig 14:**
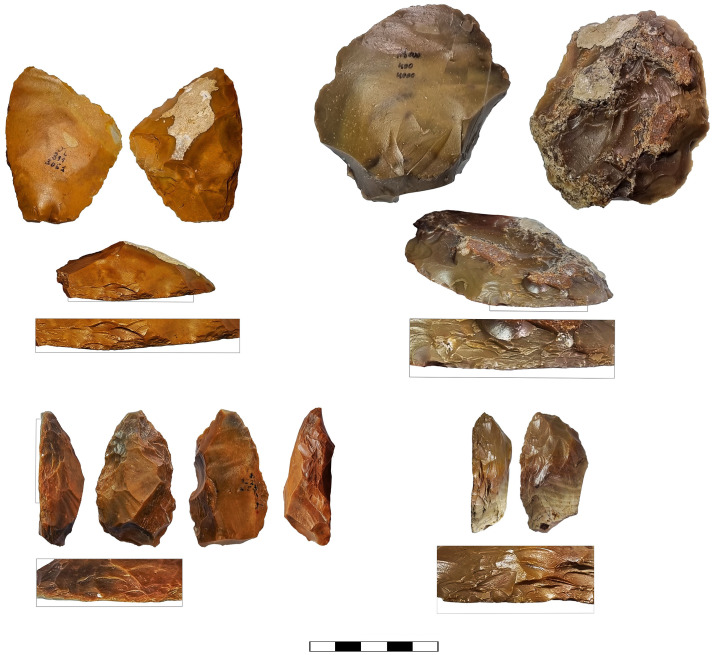
Shaped items from locality B: scraper (A, C, D, G, H), notch (B, E), Awl-Borer (F).

**Fig 15:**
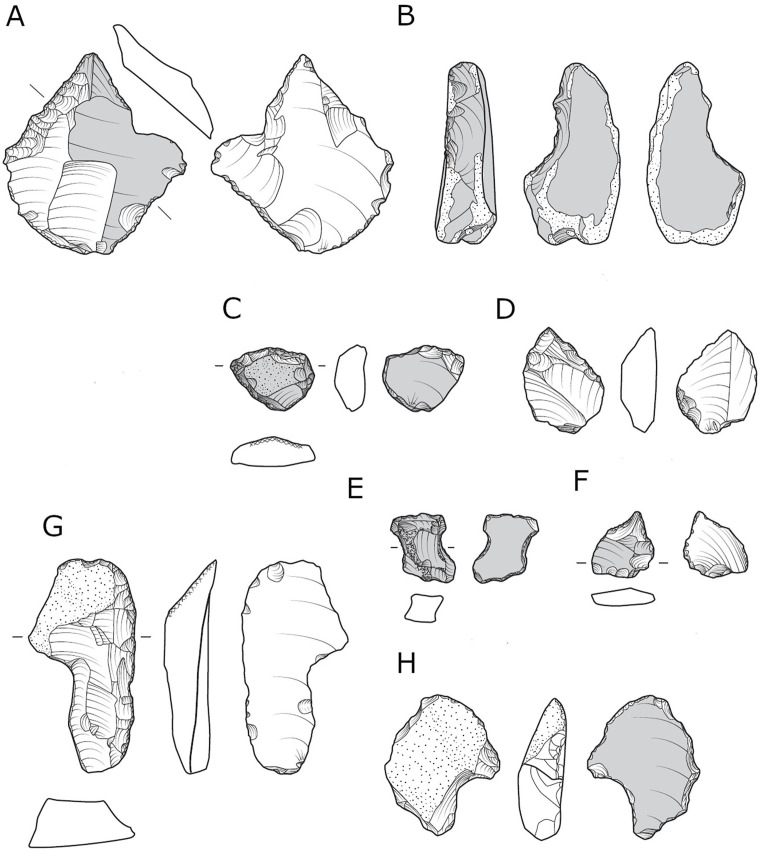
Scrapers with Quina-like retouch.

Shaped items comprise a significant part of all five assemblages (A: 24.5%; B: 29%; C: 18%; D: 38% of the *débitage*), although they are notably less common in locality E (11.1% of *débitage)*. Most of the shaped items in Jaljulia were made on flakes, whereas only up to 2% in each locality were made on laminar items, correlating to the proportions of each category among the unshaped blanks (Tables 2 and 3). Typological analysis of the shaped items (Table 9) shows that retouched flakes (Fig 13B) are the most common category in all localities, with retouched broken flakes (flakes missing their bulb of percussion) the second most common category (Table 9). It should be noted that we cannot rule out the possibility that some of the retouched flakes and flake fragments were not intestinally retouched and might resulted from post-depositional processes such as trampling, as we are dealing with an open-air site in which many of the items were indeed exposed to such processes. Therefore, the number of retouched items must be taken as an overrepresentation of this category, regardless of the fact we included only items bearing regular retouch of at least one centimeter in extent, and most items bear much more significant retouched edges. This possible over representation might also be accounted for the large proportion of shaped items out of the debitage. Notches are also quite common in all localities (Figs 13E and 14B). Side scrapers (Figs 13A, 13D, 14A, 14C, 14D, 14G and 14H) are relatively common in locality E (7.2% of all shaped items), less in localities B (2.9%), C (3.3%), and D (2.5%), and almost absent from locality A (0.4%). End scrapers are significantly less common in all localities. Noteworthy are some scrapers with retouch that bear a high resemblance to the Acheulo-Yabrudian Quina and demi-Quina scrapers (Fig 15A–15C, [53]). Thirty-four scrapers out of all localities together (16%) bear a scaled-stepped Quina-like retouch [114]. These well-known component of the European Middle Paleolithic Mousterian Cultural Complex [134,135] are characterized by scaled stepped (*Ecailleuse scalariforme*) retouch, which creates broad, sharp working edges, considered a hallmark of the Levantine Acheuleo-Yabrudian cultural complex (henceforth AYCC) [136,137]. Choppers, burins (Fig 13C), and truncations (Fig 13F) are very rare in all the excavated localities, and each is represented by a few items. Of note is the high presence of bifaces in locality E (13.4%) compared to all other localities, as well as their very low appearance in localities A (0.6%) and C (1.6%). Localities B and D are represented by the same percentage of bifaces.

**Table 9:** Composition of shaped items from all five localities.

	Locality A no.	Locality A %	Locality B no.	Locality B %	Locality C no.	Locality C %	Locality D no.	Locality D %	Locality E no.	Locality E %
**Shaped Items**	**Total**	**%**	**Total**	**%**	**Total**	**%**	**Total**	**%**	**Total**	**%**
**Retouched flake**	397	44.1%	756	37.2%	181	40.2%	1679	43.2%	55	28.4%
**Retouched blade**	14	1.6%	40	2.0%	2	0.4%	35	0.9%	1	0.5%
**Retouched** **blanks produced from COF**	11	1.2%	44	2.2%	5	1.1%	59	1.5%	3	1.5%
**Side scraper**	4	0.4%	58	2.9%	15	3.3%	96	2.5%	14	7.2%
**Scraper frag.**	0	0.0%	11	0.5%	0	0.0%	8	0.2%	0	0.0%
**End scraper**	3	0.3%	17	0.8%	6	1.3%	14	0.4%	1	0.5%
**Notch**	125	13.9%	164	8.1%	33	7.3%	376	9.7%	24	12.4%
**Truncution**	2	0.2%	22	1.1%	11	2.4%	44	1.1%	0	0.0%
**Awl-Borer**	37	4.1%	27	1.3%	8	1.8%	33	0.8%	0	0.0%
**Denticulated**	33	3.7%	52	2.6%	11	2.4%	105	2.7%	5	2.6%
**Burin**	4	0.4%	0	0.0%	2	0.4%	9	0.2%	2	1.0%
**Chopper**	7	0.8%	12	0.6%	6	1.3%	22	0.6%	7	3.6%
**Biface**	5	0.6%	127	6.3%	7	1.6%	184	4.7%	26	13.4%
**Varia**	36	4.0%	257	12.6%	43	9.6%	407	10.5%	10	5.2%
**Retouched fragment**	222	24.7%	445	21.9%	120	26.7%	820	21.1%	46	23.7%
**Total**	**900**	**100%**	**2032**	**100.0%**	**450**	**100.0%**	**3891**	**100%**	**194**	**100.0%**

**Fig 16:**
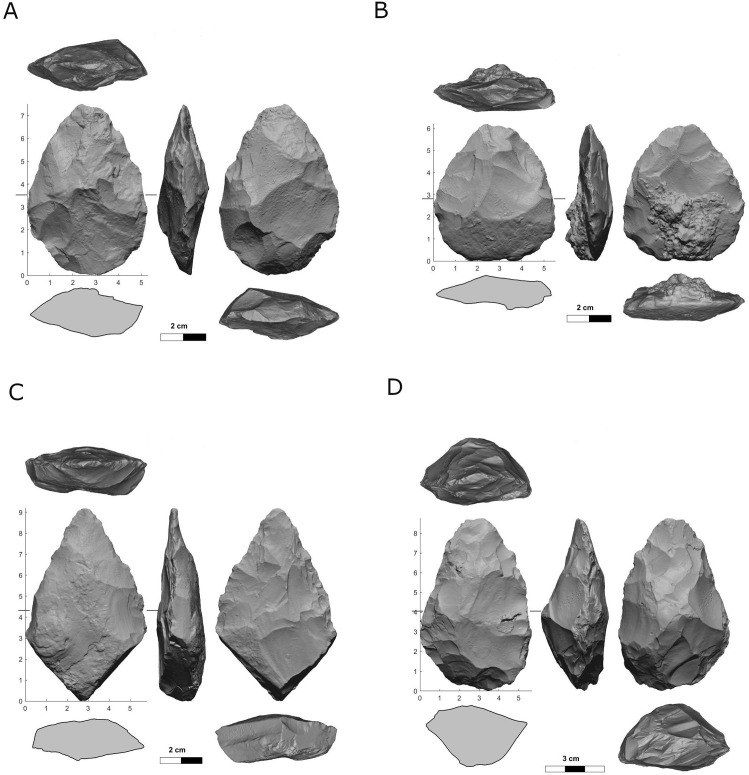
Scans of handaxes from Jaljulia localities A (A), B (B), and D (C, D).

***Bifaces*:** A total number of 373 bifaces were documented in all five localities (Fig 16) and are defined after Muller 2022 [118]. Handaxes are the most common category (A: 44%, n = 18; B: 68%, n = 77; D: 62%, n = 114; E: 38%, n = 10), the vast majority of which are complete. Only in Locality C, handaxes are the second most common, following biface roughouts. Small handaxes comprise 22% of the bifaces in Locality C, 14% in Locality D, and much lower percentages in all the other localities (Table 10). Recycled handaxes that were used as cores are common in locality E (19%, n = 5), while absent from locality C, and of lower representation at localities A, B, and D. Biface varia comprise between 12%–17% of the whole biface assemblage, the majority of which are complete, excluding locality C, where they are absent altogether.

Handaxes with preferential flake removal (Table 4), are present in all localities.

#### Tool waste.

Tool waste items are scarce in most localities, with the exception of Locality B where they constitute 3% of the *débitage*. The most common spall is the tool rejuvenation spall, which constitutes about half of the spalls in localities A and D and about one-third in localities C and E (Table 11). Second in abundance are the bifacial spalls. Notably, these items were particularly common in Locality B (Table 11), correlating to the high number of bifaces found there (Table 9). Biface thinning flakes, also termed *éclats de taille de biface* and attributed to the final stages of biface manufacture [138], are common in locality B (31.9%). The high number of biface spalls might suggest on-site reduction and modification/maintenance of bifaces. While there is a significant correlation between the numbers of bifaces and biface spalls at locality B, locality D presents a more complex picture since the number of bifaces there is very high but does not correlate with the low number of spalls. Considering the dynamic nature of stone knapping, only in rare cases could a complete reduction process be reconstructed in full in a single place and time [139–141]. Scraper spall is minimally represented in all localities (Table 11). Burin spalls are present in all localities, however in only few items (Table 11).

**Table 11:** Breakdown of tool waste in all five assemblages.

	Locality A no.	Locality A %	Locality B no.	Locality B %	Locality C no.	Locality C %	Locality D no.	Locality D %	Locality E no.	Locality E %
**Tool waste**	**no.**	**%**	**no.**	**%**	**no.**	**%**	**no.**	**%**	**no.**	**%**
**Burin spall**	2	8.0%	5	2.2%	4	19.0%	11	11.1%	1	5.0%
**Tool spall**	14	56.0%	46	20.1%	6	28.6%	59	59.6%	6	30.0%
**Biface spall- thinning flake**	2	8.0%	73	31.9%	0	0.0%	3	3.0%	3	15.0%
**Biface spall**	5	20.0%	54	23.6%	4	19.0%	8	8.1%	3	15.0%
**Scraper spall**	0	0.0%	13	5.7%	0	0.0%	0	0.0%	2	10.0%
**Varia**	2	8.0%	38	16.6%	7	33.3%	18	18.2%	5	25.0%
**Total**	**25**	100.0%	**229**	**100.0%**	**21**	**100.0%**	**99**	**100.0%**	**20**	**100.0%**

## Discussion

The analysis of five Late Acheulian assemblages from Jaljulia reveal characteristics of typical Levantine Late Acheulian industries in all localities, bearing similarities to other assemblages from Late Acheulian sites such as Revadim, Kefar Menachem West, Berekhat Ram and Eyal 23 [75,79,142–145].

However, alongside the underlying techno-typological similarities, these analyses showed intriguing variabilities between the studied assemblages, reflected in tool frequencies and abundant typologies, as well as in core typology and in the frequencies of prepared cores and their distinguished products. These differences provide important insights about potential changes in focus and type of activity conducted in each of the studied localities and might reflect on diachronic adaptations in Late Acheulian industries.

### Similarities and differences in Core reduction sequences

The current lithic analysis reveals that core reduction sequences at the site include three main trajectories: 1. production of large, medium, and small-sized flakes from ‘regular’ cores, using one-, two-, or multiple-striking platforms; 2. production of predetermined items from prepared cores; 3. production of very small flakes from core-on-flakes, some which represent a mode of lithic recycling.

Notably, the analysis presented here suggests that the dichotomy between prepared and unprepared cores should not be so strict, since the high number of CTEs of different types clearly suggests that some ‘regular’ cores were shaped and maintained to some extent during core reduction. One example is the presence of core tablets and ridges, maintenance items that are not related to prepared core reduction, and were found in all five assemblages (Table 6). Another example is the high frequency of flakes with modified bases (Tables 2 and 3).

One of the primary sources of variability between the Jaljulia assemblages is the frequencies of prepared cores and the abundance of their products. Based on our detailed analysis of the cores, associated waste, and resulting products from Jaljulia Locality B, we term these cores *proto-Levallois* to highlight their technological and conceptual proximity to the later, fully developed Levallois method. These demonstrates a high degree of planning and core preparation aimed at producing predetermined blanks, key features that are central to Levallois technology. Our previous study showed that such capabilities were already within the reach of late Acheulian populations in the Levant, and as such, the term *proto-Levallois* appropriately reflects the initial appearance of these behaviors and their role in the emergence of Middle Paleolithic technological systems [29]. Localities A, C, and E show relatively low representations compared to localities B and D, where a higher percentage of prepared cores was observed (Table 12). In addition to the many prepared cores that were found at Jaljulia, a noteworthy number of flakes produced from prepared cores, as well as related CTEs, were observed in all assemblages (Tables 2 and 3). Although they constitute only about 1% of the total assemblage, the presence of 99 such items from Area B and 21 in Locality A renders this component meaningful within the broader technological context.

**Table 12:** Comparison of prepared cores from other Acheulian sites: Revadim [77,145,146]; Holon [147]; Berkhat Ram [143] and Gesher Benot Ya`aqov [7].

Site/Locality	Percentage of Prepared Cores from all Core types	Notes
Jaljulia – Locality A	4.9%	
Jaljulia – Locality B	16.7%	
Jaljulia – Locality C	5.3%	
Jaljulia – Locality D	10.9%	
Jaljulia – Locality E	4.7%	
Revadim – Layer C5	25%	
Revadim – Locality B	8–9%	
Revadim – Layer C3 West	4%	
Revadim – Layer C East	12.98%	Defined as “cores with two surfaces perpendicular to each other with hierarchy“.
Berekhat Ram	64.8%	Note: smaller sample size.
Holon	3.2% (or 19.3%)	19.3% refers to ‘cores with hierarchy,’ potentially corresponding to the definition of prepared cores.
Gesher Benot Yaʻaqov	5.2–17.3%	Variability observed across different layers.

This reflects a complete reduction sequence of prepared cores knapping, maintaining, and reshaping all taking place, to some degree, at the site. Notably, locality B, the youngest locality in Jaljulia that has been studied thus far, shows the highest percentage of prepared cores. Similar variability is shown in other late Acheulian sites. At Revadim, an intra-site variability is reflected as in the assemblage of Layer C5, prepared cores constitute a quarter of all the cores [75], while in locality B, Layer C3 West, prepared cores appear at lower frequencies (Table 12; [145,77]). In locality C East of Revadim prepared cores, defined as “cores with two surfaces perpendicular to each other with hierarchy”, comprise 12.98% of all cores [146].

In the assemblage from Berekhat Ram, prepared cores constitute ca. 65% of the cores [143], whereas in the assemblage from Holon, they represent only 3.2% (or 19.3% for cores with hierarchy, which might correspond to our definition of prepared cores) [147]. These are indeed great discrepancies in the representation of prepared cores between late Acheulian sites, and await further detailed studies. We note, that for these sites we trust published figures, and to some extent differences in definitions and observations between researchers could also account for some distortions in numbers. Hopefully, an updated analysis of all late Acheulian prepared cores from sites in the southern Levant would shed new light on this intriguing phenomenon.

Notably, a similar intra-site variability of prepared cores was also noted in the Middle Acheulian site of Gesher Benot Ya‘akov, ranging between 5.2% and 17.3% in each layer [4].

These numbers suggest that while prepared core technology was familiar to Acheulian knappers, the abundance of its application and use was inconsistent, and probably related more to site function or the type of activity conducted rather than to diachronic aspects. This is reflected well in the inter-variability of Jaljulia: Locality D, dated the oldest of the Jaljulia localities, presented the second-highest abundance of prepared cores, somewhat correlating to the frequencies of Locality B at Revadim. On the other hand, the youngest Locality B, while presenting the highest frequencies in Jaljulia, still demonstrates lower dominance of prepared cores compared to Layer C5 in Revadim and even to some of the Middle Acheulian Layers at GBY (Table 12).

However, the comparison between sites indicates that some of the variability might be related to different terminologies applied by different research groups, resulting in different approaches to what is included in the ‘prepared cores’ category. Furthermore, diachronic trends might be reflected in the dominance of specific sub-types (e.g., Discoid or proto-Levallois). These are subjects for future research.

The diversity of core reduction sequences utilized in Jaljulia, including the variety of core maintenance items, reflects a complex world of knapping for the inhabitants of the site.

One prominent example of such complexity is the presence of Handaxes with preferential flake removals, reflecting a technology that is present in many Acheulian sites throughout the Old World [48,56,65,78,48,120]. In a recent paper, we offered to view these items as evidence for the existence of cumulative culture as early as the end of the Lower Paleolithic [56]. Cumulative culture refers to the long and multi-participant process of an idea that progresses and develops, resulting in technological innovations [148–152], sometimes termed the ratchet effect [148,149,152].

### Shaped items

Another point of variability between the Jaljulia assemblages is marked by the frequencies and typologies of the shaped items. In Localities B and D, these constitute a significant part of the assemblage (12.3% and 16.1%, respectively), while in Localities A and C they are less abundant (7.5% and 6.6%, respectively). The lowest frequency of shaped items was noted in Locality E (3.4%; Table 3). These differences are intriguing as they might reflect composite processes of post-deposition, as well as differences in focus between localities. Thus, the low tool frequencies in Locality E might be related, at least in part, to its location on a hill overlooking the fluvial plain in which the other localities were deposited. This would have prevented edge damage caused by fluvial activity, which is sometimes difficult to distinguish from an intentional retouch. In addition, the thin archaeological horizon exposed at Locality E implies a less intensive occupation of that locality, when compared to Localities B and D (Table 1), which would result in less edge damage caused by trampling. This line of thought is further supported by the relatively high frequencies of ’formal tools’ (i.e., scrapers, bifaces, chopping tools) in Locality E, compared to the other analyzed localities where high dominance of retouched flakes was noted (Table 9).

On the other edge of the spectrum, the high frequencies of shaped items in Localities B and D, which present a good state of artifact preservation, could reflect a palimpsest of occupations in those localities on the one hand, and a possible focus on more ad hoc activities compared to Locality E.

In Localities A and C, the relatively low tool frequencies correlate to a low density of artifact deposition and to the evidence of intense fluvial activity, which affected artifact preservation and likely caused some post-depositional movement [113].

Despite the variability between localities, the shaped items frequencies from the Jaljulia assemblages correlate well with other Late Acheulian sites in the region. In Revadim, shaped items comprise 4.9–12.9% of each assemblage [75], whereas at Kfar Menahem West they constitute 7.8% [142], and at Berekhat Ram only 5.9% [143]. The site of Holon presents the closest parallel to Jaljulia, Locality D, showing a tool frequency of 15.4% of the assemblage [147].

Another key part of the Jaljulia industry is the presence of biface tools. These (Table 10) reflect a great deal of variability among the five assemblages: Localities A and C have a relatively low representation (A: 0.6%; C: 1.6%) compared to localities B, D, and E, where a higher percentage of bifaces was observed (B: 6.3%; D: 4.7%; E: 13.4%). These differences could be explained by the differences in preservation and location of the Localities as discussed above, implying that localities B, D, and E are closer to reflecting the original abundance, whereas Localities A and C might have been depleted by fluvial activity and therefore in these localities, bifaces are underrepresented. Nevertheless, other Late Acheulian sites also present variability in biface dominance, implying that the changes in frequencies might relate to site function and to different activities conducted in each locality. Thus, at Revadim bifaces constitute 0.02–4.3% of the shaped items [75,79,145,146], and at Berekhat Ram, 1.9% [143] whereas at Kfar Menahem West, not a single handaxe was found [142]. The Late Acheulian site of Holon presents the highest biface abundance, where these items represent 23.4% of the shaped items [147].

### Intra-site refinement variability

A broad look at the technological skill level, as reflected by the different trajectories of the lithic industry at Jaljulia raises an interesting point involving the dissonance between the high-level investment, complexity, and depth of planning of the proto-Levallois reduction sequence, as opposed to the much less refined Jaljulia handaxes. In a 3D analysis of the Jaljulia handaxes, 260 from localities A, B, C, and D were studied for criteria such as center of mass, scar density, surface locality, edge length, and geometric morphometrics [82]. They concluded that the Jaljulia Handaxes were relatively small and thick and highly variable in shape, indicating their manufacturing under a non-strict *chaîne operatoire.* The oldest locality (Locality D) contains the most refined and smooth-outlined handaxes, whereas the younger localities showed less refined handaxes [82]. This trend of change that we see in Jaljulia is present on other sites. A recent study, including the analyses of handaxes from several Levantine sites, showed that the Middle Acheulian assemblages presents higher values of morpho-technological aspects associated with craftsmanship than some of the Late Acheulian assemblages [118].

In Jaljulia, the decline in investment in handaxe manufacture correlates to an increase in the abundance of prepared cores technologies (cores, CTEs and end products), as reflected by a rise in their numbers between the oldest locality D and the younger locality B (Tables 2, 3 and 6).

In addition, recent work on the Jaljulia raw materials showed that the Jaljulia knappers possessed a nuanced understanding of the raw materials available in their surroundings [52]. Four groups of artifacts were classified into flint types: a general sample, bifaces, “regular” cores with one/two striking platforms, and prepared cores. While locally available flint was predominantly used, specific flint types were selectively exploited for the production of particular tools, based on their properties. The analysis of 60 bifaces from localities B and D found brecciated flint types to be especially dominant in producing these tools, while other materials such as fine-textured homogenous flint types were more suitable for the Proto Levallois, where more control over the end product was needed. Therefore, this work indicated that the Jaljulia inhabitants purposefully selected distinct types of flint for the production of specific tools, underscoring a deliberate and non-random approach to material utilization.

Based on these studies, we suggest that these differences in handaxe finesse, prepared core frequencies, and raw material use involved the deliberate choices of the knappers, reflecting growing investment in prepared core technologies at the expense of handaxe shaping. Thus, the slow implementation of the concept of the Levallois method may have moved the center of attention, in terms of knapping investment, from the older, long-lasted handaxe to the new innovative technology. Changes and shifts in trends of technological advancement or decline are influenced by a wide range of factors, some rooted in the availability of raw materials and the changing functional needs of the group, as a way of ecological adaptation. Others stem from social processes (and in later periods also political [153]) that shape patterns of technological development and use that influence the cultural, economic, and social contexts within which innovation and the adoption of new technologies occur. We argue that adopting a broad perspective on the emergence and transformation of technologies, as part of a larger picture that encompasses influences from various aspects of life, is essential. In a previous study, we attempted to link the advancement and emergence of advanced core technology, the proto-Levallois in the Levant with significant changes in fauna, particularly the initial disappearance of elephants from the region [29]. We propose that external factors influence not only the appearance and decline of technologies but also the decisions regarding where to invest technological efforts resulting in its refinement and reflecting what is considered significant or central at a given time.

An interesting example of a decline in investment in the lithic industry can be observed, albeit at a much later period, in the flint industry at Nahal Zehora. There, it was suggested that the introduction of pottery as a completely novel technology, alongside political considerations, may have contributed to a decrease in investment in the older industry, and to an increasing interest in the new one [153].

Regarding the Jaljulia assemblages, these trends are more likely reflect human choice as to where to invest; in this case, leaning towards the new innovative technology. In addition, we suggested that the elephant decline toward the end of the Lower Paleolithic may have acted as a catalyst for the development of the Levallois technique during this phase. The end of the Lower Paleolithic may have acted as a catalyst for developing new light-duty butchery implements which were used upon smaller-sized prey. As early humans witnessed the disappearance of their preferred prey, new technologies were introduced while the attention for other trajectories shifted, sometimes simultaneously. The Levallois technique used for the production of very sharp flakes with regular edges might have been the peak of the process [29].

The different tool technologies and the range of skill levels indicated in the Jaljulia assemblages may also suggest that no linear progression is reflected in the chrono-typological aspect. Intra-site variability of skill levels between technologies is far more complex than drawing a simple linear picture. Each trajectory must be evaluated on its own while taking into consideration the relationship between the different trajectories.

### Aspects of cultural continuity

The scarcity of large scale excavated Late Acheulian sites in the Levant, together with the complexity of the Late Lower Palaeolithic, particularly regarding the relationship between the Late Acheulian and the Acheulo Yabrudian Cultural Complex, makes the case of Jaljulia especially intriguing. The comparison of Jaljulia assemblages to other later Acheulian sites, presented in the current study, is crucial for an understanding of the Late Acheulian and its relationship with the following Levantine Acheulo-Yabrudian and Middle Paleolithic Mousterian.

**Fig 17:**
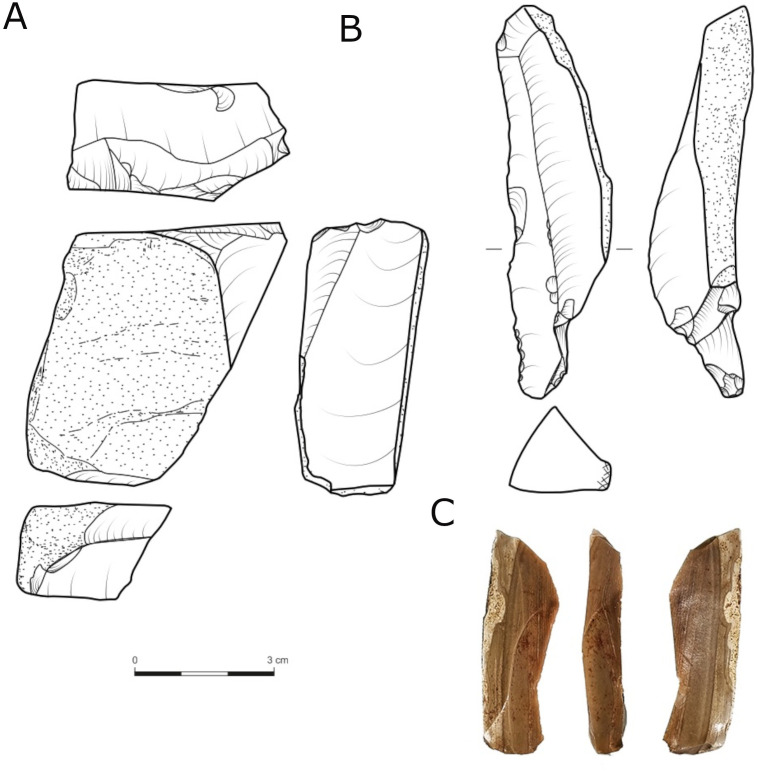
Blade core (A) and unretouched blades (B,C).

From a wider geo-chronological perspective, the vicinity of Late Acheulian Jaljulia to the Achelo-Yabrudian site of Qesem cave merits the examination of techno-typological similarities and differences between lithic industries. A significant hint towards similarity lies in the relatively high number of blades (Fig 17), shaped or unshaped, revealed in Jaljulia, compared to other Acheulian sites in the region [e.g., 75,147].

The higher number of blades in Jaljulia is found in the youngest analyzed locality, Locality B, whereas Locality D, the oldest locality in Jaljulia [113], has a relatively low number of blades (Tables 2, 3 and 9), suggesting a pattern of growing frequencies of blade production through time. As a working hypothesis, this might be viewed as a possible precursor of the rich Achaeuleo-Yabrudian blade industry [154]. In addition, demi-Quina scrapers were found at some of the assemblages from Jaljulia, typically considered the hallmarks of the AYCC in the Levant [53,137,155,156] and a well-known component of the European Middle Paleolithic Mousterian [134,135], though well refined examples of scrapers have also been identified at various British Late Acheulian sites [34,157–160]. Their appearance in Jaljulia might represent the first steps in the distribution of this technological innovation and may be related to changes in butchery behavior and new preferred prey [114,136]. The possible continuity pertains not only to the technological level but also to the possibility of an ontological-cosmological connection. It appears that the flint types used to produce these scrapers both in Jaljulia and in Qesem cave originated from a similar locality, likely a locality where deer were hunted and later butchered using these scrapers. The onset of Quina-like technology in Late Acheulian Jaljulia may suggest practical and ontological continuity between the two Late Lower Paleolithic entities [114,136].

However, while the existence of blades and Quina scrapers at Jaljulia might imply some resemblance to the Acheulo-Yabrudian at Qesem Cave, it is the difference between the industries that stands out. Most prominent is the presence of the prepared cores technologies at Jaljulia, and their absence in Qesem Cave. Qesem Cave contains two of the three known AYCC components: a blade-dominated industry known as Amudian, and an industry dominated by Quina (and demi-Quina) scrapers, known as Yabrudian. These two industries interchange throughout the sequence [112]. The possible chronological overlap between the youngest activity phases in Jaljulia and the human activity in Qesem Cave [113], makes these differences worth investigating, as the inhabitants of these two geographically adjacent sites, produced very different lithic industries (excluding some specific points of resemblance). This intriguing relationship between the traditional Acheulean Juljulia assemblages and the innovative Qesem industries is not a straightforward scenario of continuation or replacement, nor does it fall clearly under the definition of cumulative culture. One example is the roots of the Levallois method that are claimed to be a part of the Jaljulia assemblage [29], which, if a linear developmental approach is assumed, then disappear or drastically decline in the Achuelo-Yabrudian [60,73,112,161,162,156], only to re-appear at a peak of abundance in the Middle Palaeolithic Mousterian [163]. This example highlights the inherent complexity of technological development, demonstrating the impossibility of framing every technology as a linear progression of continuity and improvement in a cumulative manner. Additionally, this may reflect a group-scale human choice. That is, the Qesem Cave knappers may have known and understood the Jaljulia knappers’ technical skills and way of doing things, and had chosen to act differently. A previous study suggested that the handaxes in Qesem Cave are not an integral part of the lithic industry on site. Rather, they were most probably collected from close-by Acheulian sites [52].

The possibility that Qesem Cave inhabitants’ collected “old”, ready-made, handaxes and stone balls implies their familiarity with some of the most characteristic elements of the Acheulian culture [52,164]. This pattern of behavior indicates an awareness and appreciation of the antiquity of these old, knapped artifacts [52,165], but foremost, it indicates familiarity with the industries that preceded theirs.

## Conclusions

The optimal conditions around Jaljulia led human groups to revisit the landscape throughout the late Lower Paleolithic period, creating the different excavated localities presented in the paper.

The general similarity in the major techno-typological features of the five assemblages can indicate that the knappers at these different localities at Jaljulia practiced the same technological traditions, thereby strengthening our view of these localities of activity under the same site.

The results allow some conclusions to be drawn regarding the cultural evolution in the Late Acheulian. A gradual, but not definitive, decrease in handaxe refinement, alongside a growing investment in prepared core technologies may have taken place during the final phase of the Acheulian. On the other hand, the early appearance of technological innovations, starting from the oldest Jaljulia locality, suggests a local and autochthonous development at the site that took shape during many millennia of consecutive visits to this preferable locality.

The variability among Jaljulia assemblages can thus reflect some chronological aspects or different activities performed at each locality but does not seem to be related to different technological perceptions of Jaljulia’s knappers.

In the broader context of the Late Acheulian in the Levant, the Jaljulia assemblage, as presented in this study, enhances our understanding of the cultural and technological variability characterizing this period. On the one hand, it exhibits technological behaviors consistent with other contemporaneous Late Acheulian assemblages while on the other, it reveals notable similarities with later developments, both with the Acheulo Yabrudian Cultural Complex, as reflected in the early appearance of specific technologies such as scraper production and blade manufacture, and with Middle Palaeolithic technologies, as seen in the emergence of prepared cores. These characteristics position Jaljulia as an important reference point for examining the complex technological and cultural processes that marked the end of the Lower Palaeolithic in the Levant.The current lithic analysis makes an important contribution to the research as an additional large-scale database that can be used for further, more specific analysis in the future. In addition, it notes some interesting patterns of human behavior, that emphasize the place of human choice in the trajectory chosen for manufacture and in the amount of investment in each of them. These allow for a better understanding of the late Acheulian lithic industry at Jaljulia, reflecting human behavior during the late Lower Paleolithic characterized by both continuity and innovation.

## Supporting information

S1 File Supporting information 1. Methodology. (DOCX)

S2 File Supporting information 2. Inclusivity in global research. (DOCX)
