# Correction: Late Acheulian stone-working by the riverbank: Patterns of continuity and change reflected in Jaljulia lithic assemblages, Israel

**DOI:** 10.1371/journal.pone.0343782

**Published:** 2026-02-24

**Authors:** Tamar Rosenberg-Yefet, Aviad Agam, Ella Assaf, Bar Efrati, Yafit Kedar, Vlad Litov, Maayan Shemer, Ran Barkai

The images for Figs 14 and 15 are incorrectly switched. The image that appears as Fig 14 should be Fig 15, and the image that appears as Fig 15 should be Fig 14. The figure captions appear in the correct order. The authors have provided a corrected version of the figures here.

**Fig 14:**
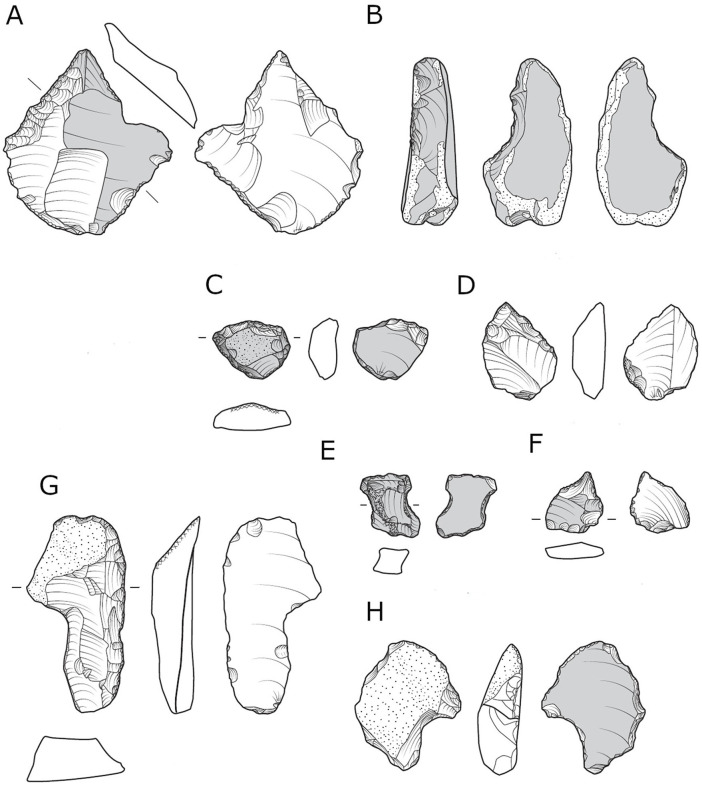
Shaped items from locality B: scraper (A, C, D, G, H), notch (B, E), Awl-Borer (F).

**Fig 15:**
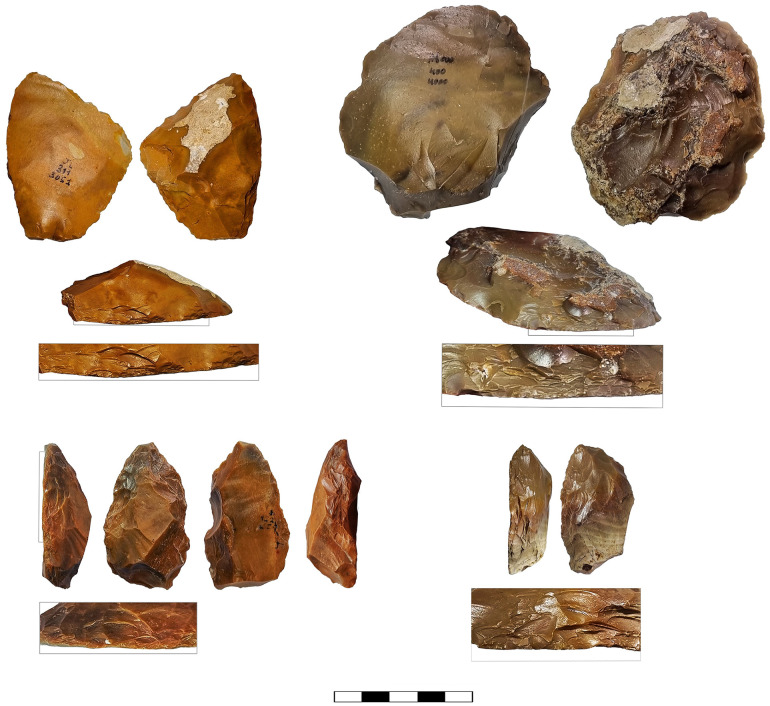
Scrapers with Quina-like retouch.
