# Supplementary material for: Late Acheulian stone-working by the riverbank: Patterns of continuity and change reflected in Jaljulia lithic assemblages, Israel
Source: PLoS One. 2025 Dec 29;20(12):e0338540. doi: 10.1371/journal.pone.0338540 (PMC12747397; doi:10.1371/journal.pone.0338540)
Supplement: S1 File — (DOCX) [file pone.0338540.s001.docx]

**Methodology**

**Cores**

Cores were defined as items bearing at least one striking platform from which at least one flake was removed. We also included in the core category flint nodules/cobbles from which a single “testing flake” was removed, either from an unprepared/natural platform or following the preparation of a striking platform. These items are termed here ‘tested pebble/cobbles’.

In addition, the analysis presented here applies a distinction between “regular” cores with one-, two- or multiple- striking platforms, which show minimal or no indication for intentional shaping and maintenance of the production surface, and cores that do indicate such shaping and maintenance. The latter are referred here as ‘prepared cores’.

Prepared cores were further divided into three sub-types: Prepared cores (general), Proto-Levallois, and discoid cores .

Prepared cores (general) are cores that did not meet the criteria of the proto-Levallois cores and the discoid cores and were defined in the spirit of Debénath and Dibble [137], who defined them as “*a number of technologies … in which the core was intentionally shaped or prepared in such a way as to predetermine the shapes of flakes taken from it*.” All cores under that definition are fully or partially centripetal in their design, in contrast to one, two, or multiple- platforms cores, which are more orthogonal. Therefore, all prepared cores are characterized by two surfaces and a plane of intersection, although their exact role in the reduction sequence varies between knapping methods, and presence or absence of hierarchy between them (i.e., Proto-Levallois or discoid).

Scholars are not in consensus regarding the terminology of cores bearing conceptual resemblance to Levallois cores, which originate from pre-Middle Paleolithic contexts [23]. Here we follow the definition proposed by Picin [138], whereby proto-Levallois cores are defined by a division of the volume is into two hierarchical surfaces: A striking platform and A production surface, with a plane of intersection separating the two. The plane of intersection is delineated by a partial or complete bifacial ridge. Striking platforms are usually roughly prepared, and lateral and distal convexities of the production surface are roughly configured by either *débordant* flakes or by preparational flaking of the core circumference [138]. In our studies of late Acheulian assemblages, we did not define such cores as fully fledged Levallois, although some do represent a more developed technological stage and meet all of Boëda’s criteria [129]. The main technological difference between typical Levallois cores and the ones presented here concentrates on a less strict application of the criteria. For instance, core circumference is not always prepared fully and lateral and distal convexities of the flaking surface are sometimes only roughly designed in Proto-Levallois cores. For a detailed description of methodological issues concerning Proto-Levallois cores from the Levant see [78].

The definition of discoid cores follows the guidelines set out by Terradas [139], who sees the discoid flaking method as “*core exploitation in order to obtain various numbers of flakes by centripetal, recurrent and usually bifacial organization of removals. This exploitation proceeds by maintaining a strong stability of the volumetric concept of the core, which requires little specific preparation of its striking platforms and flaking surfaces. The core resulting from this sort of exploitation has an oval shape and a biconvex asymmetric section*” [139].

A collection of studies published in a volume edited by M. Peresani [140] showed indication of broad variability between discoid cores from different chronological and geographical distributions, leading researchers to distinguish between *sensu stricto* and *sensu lato* definitions of discoid cores [141]. The term discoid *sensu lato* better describes cores that exhibit a hierarchy between the two surfaces, whereas *sensu stricto* describes cores that lack such hierarchy and in which the direction of production is secant to the plane of intersection of the two surfaces [141]. Here, the definition of discoid cores refers to cores that exhibit hierarchy between the two surfaces, which could be interchangeable.

In addition to this group of cores, we defined items that were removed from these cores during the reduction sequence as CTEs of prepared cores. In order to clarify, CTEs refer to all items that were removed from the core with the aim of shaping or maintaining it throughout the reduction sequence. This group includes *Entame* flakes, ridges, core tables etc. Within this group, we identified a more specific group of waste items resulting from the shaping process of prepared cores specifically, termed here prepared core CTEs. These items were removed from prepared cores typically involving the removal of the intersecting ridge between the two platforms, most probably in an effort to maintain core convexities throughout the reduction sequence. These items do not fully conform to the definition of specific CTEs such as: *éclats débordants*, *éclats débordants a dos limité* and *éclats outrepassés*, which are items with a more narrow definition that relates to its specific location on the core. For a detailed description of these items with sub-division into different *débordants types* from locality B see Rosenberg-Yefet et al. 2022.

For a detailed description of all prepared cores and their definitions from Jaljulia, see [78].

Cores-on-flakes/Flaked flakes (henceforth COF) are presented here separately from the general ‘cores’ category, in order to allow comparison with other Acheulian assemblages. COF (following [95, 142] are flakes displaying the removal scars of one or more smaller flakes, either on the ventral or the dorsal faces.

The small flakes detached from COF are termed here blanks produced from COF. They are characterized by two flat and smooth faces (ventral and ventral-like) and, in some cases, also a straight, thin profile [22, 24, 96, 114, 143]. Small flakes were further subdivided according to their place of removal from the parent flake.

For a detailed definition of all categories of products of COF, see [26].

An additional sequence of recycling, is reflected in the use of handaxes as cores for the production of predetermined blanks, termed here handaxes with preferential flake scars [87]. In the assemblages from Jaljulia, some of these items were subjected to detailed analyses based on the following characteristics: 1. Preparation of a striking platform? prior to the detachment of the preferential flake; 2. The presence of patina differences between the bifacial shaping/resharpening scars and the preferential flake/s scar/s; 3. The preferential scar morphology is different from the bifacial shaping scars; 4. The preferential flake scar is cutting the previous bifacial scars; 5. The preferential flake detachment took advantage of handaxe convexities; 6. The preferential flake detachment removed a part of the bifacial ridge. While counted as handaxes (Table 10), these items are also described here in the chapter describing reduction sequences.

For a detailed description and discussion of handaxes with preferential flake scars in the Levantine late Acheulian, see [13].

**Debitage**

The analyses presented here applied a division of the lithic assemblage into two broad categories: technologically determined blanks (i.e., that could be linked to a specific reduction sequence such as proto Levallois, discoid, prepared cores flakes, flakes produced from cores on flakes); and technologically undetermined blanks (i.e., flakes and blades that could not be ascribed to a specific reduction sequence).

The following terminology was used to describe specific debitage categories: Primary flakes are items with a bulb of percussion and at least 30% cortex on the dorsal face; Modified base flakes (MB flakes) are flakes with a bulb of percussion and a modified base that was shaped prior to its detachment from the core; non-modified base flakes (NMB flakes) are flakes with a plain base.

Modified bases were further divided into faceted, partially modified, and dihedral. Faceted refers to bases showing several preparation negatives (facets) and are varied morphologically. Due to the high variability of modification, we separated flakes having only part of the striking platform modified and termed these partially modified. Dihedral refers to bases showing the negatives of two previous removals, separated by an arris [81]. The bulb is exactly in the point of meeting between two removals. Punctiform flakes have a tiny, point-like, striking platform. *Chapeau de gendarme* bases are defined thus: “*the profile of this very distinctive butt should be looked at face-on; while this type of butt is common in Levallois debitage (for a good, preferential impact point), it occurs during every period, irrespective of the methods applied*” [82].

Liped flakes are items usually related to the use of soft hammerstone and are characterized by having a slight projection of the ridge formed by the butt ad the lower face [82]. Micro-flakes are defined as complete items with a bulb of percussion, which are smaller than 1.5 cm. Prepared core flakes are items removed from prepared cores of one of the methods (proto-Levallois, discoid, or prepared core general), and seems to be desired end-products of the flaking process rather than aimed to shape or maintain the core during reduction.

Special waste items were detached from tools during modification, reshaping, or resharpening (e.g. bifacial thinning flakes, burin spalls, scraper rejuvenation flakes, and more).

Shaped items (tools) are defined as blanks and nodules that were modified by retouch, flaking or burin like removal.

Retouched flakes are shaped tools characterized by intentional retouch along one of their edges, which do not conform to the typological criteria of other flaked tool categories.

**Debris**

Chunk flakes are items with a clear ventral face and no bulb of percussion. Chunks are defined as produced items that lack a clearly identifiable ventral surface. Chips are items with a clear ventral face and no bulb of percussion that are smaller than 1.5 cm.
